# Fruquintinib saddles tumor immune tolerance by curbing pro-tumoral immature myeloid cell populations

**DOI:** 10.3389/fimmu.2025.1699980

**Published:** 2025-12-11

**Authors:** Lucía Suárez, María Martínez-Azcona, Irantzu Serrano-Mendioroz, Leticia Fernández-Rubio, María Esperanza Rodríguez-Ruiz, Ana Rouzaut

**Affiliations:** 1 Department of Biochemistry and Genetics, University of Navarra, Pamplona, Spain; 2 Program of Immunology and Immunotherapy, Center for Applied Medical Research (CIMA), Pamplona, Spain; 3 Departments of Immunology-Immunotherapy and Radiation Oncology, Cancer Center Clinica Universidad de Navarra, Pamplona, Spain; 4 Centro de Investigación Biomédica en Red de Cáncer (CIBERONC), Madrid, Spain; 5 Institute for Health Research (IDISNA), Pamplona, Spain

**Keywords:** myeloid progenitors, immune tolerance, VEGFR3/FLT4/CD310, tumors, fruquintinib

## Abstract

**Introduction:**

Myeloid-derived cells, particularly immature populations and tumor-associated macrophages, play a pivotal role in establishing immune tolerance and suppressing antitumor responses, thereby promoting cancer progression. Macrophage-derived lymphatic endothelial cell progenitors (M-LECP) are a population of VEGFR3/FLT4/CD310^/^bone marrow-derived myeloid precursors that contribute to tumor lymphangiogenesis, metastases, and resistance to chemotherapy through activation of TLR receptors. In this context, we investigated the effects of fruquintinib, a selective oral VEGFR3/FLT4/CD310 inhibitor with high affinity for VEGFR3/FLT4/CD310, on tumor growth and size on colorectal (MC38, CT26) and breast (4T1, E0771) tumors. We next focused on its capacity to reshape the myeloid immune compartment in the syngeneic MC38 murine colorectal cancer model.

**Results:**

Fruquintinib treatment significantly inhibited primary tumor growth, angiogenesis, and metastases in murine models of breast (4T1 and E0771) and colorectal (CT26 and MC38) cancer, respectively. Importantly, treatment with fruquintinib remodeled the tumor immune microenvironment of MC38 tumors by increasing the percentages of CD4^+^ and CD8^+^ T cells and selectively reducing myeloid cells, particularly CD11b^+^ populations of tumor-associated macrophages (TAMs) and type 2 dendritic cells. Notably, we observed similar effects in the bone marrow. In addition, treatment with fruquintinib reduced the population of bone marrow M-LECP, indicating a systemic impact. *Ex vivo* differentiation of macrophage-derived lymphatic endothelial cell precursors (M-LECP) from bone marrow cells was inhibited by fruquintinib and shifted towards pro-inflammatory phenotypes secreting IL-6, Il-1β, and less IL-10. Moreover, fruquintinib improved tumor responses to nab-paclitaxel and inhibited nab-paclitaxel-induced *ex vivo* differentiation of M-LECP. Finally, *in silico* analysis of VEGFR3/FLT4/CD310 expression in samples from cancer patients revealed higher expression of VEGFR3/FLT4/CD310 in metastatic tumors, as well as an association between VEGFR3/FLT4/CD310 expression and poorer patient survival.

**Conclusion:**

Overall, our findings offer new insights into the contribution of VEGFR3/FLT4/CD310 inhibition to restoring a pro-inflammatory tumor myeloid compartment and suggest M-LECP cells as candidate fruquintinib targets to overcome immunosuppression in tumors.

## 1 Introduction

The lymphatic vessels serve as the natural conduits for antigen-presenting cells (APCs) to reach the lymph nodes and generate a proper immune response, in addition to their role as the physiological drainage system for peripheral macromolecular clearance (1). In healthy adults, the lymphatic vessels are quiescent except during the female menstrual cycle and in wound healing. In contrast, intense lymphangiogenesis occurs in several pathologies such as chronic inflammation, transplant rejection, and cancer to control tissue edema and immune infiltration (2).

The activation of the VEGFR3 (also named FLT4 or CD310 pathway by its ligands VEGFC and VEGFD is the most relevant signaling pathway controlling lymphangiogenesis. The overexpression of VEGFC in tumors has been associated with lymphatic proliferation and enlargement, increments in metastases, and worse prognosis in cancers such as melanoma (3), breast (4), colorectal (5), and gastric cancer (6). Furthermore, tumor stromal cells such as macrophages (TAMs) (7) and cancer-associated fibroblasts (CAFs) can secrete VEGFC and other lymphangiogenic factors (8) that contribute to the lymphangiogenic sprout.

In addition to their role in tumor cell transit, lymphatic vessels contribute in different ways to the re-education of the microenvironment towards an immunosuppressive phenotype for example, through regulation of effector T cell populations (9), altering antigen presentation (10) or the impairing dendritic cell maturation (11–13).

Although the expression of VEGFR3 has been classically associated with lymphatic endothelial cells, this receptor is also expressed in some TAMs and in myeloid-derived lymphatic endothelial progenitors (M-LECPs). In fact, VEGFR3 signaling in macrophages influences their plasticity from pro-inflammatory (M1-like) to anti-inflammatory (M2-like) phenotypes (14) and M-LECPs contribute to tumor lymphangiogenesis and immune modulation making them a unique target to impair lymphatic metastasis and myeloid-driven immunosuppression (15). For this reason, specifically targeting VEGFR3 in tumors has emerged as a promising target to inhibit tumor growth (16).

Multi-tyrosine kinase receptor inhibitors are currently in use in the clinic that target the VEGF pathway. Among them, Regorafenib (BAY 73-4506, Stivarga^®^) an oral multi-kinase inhibitor targeting VEGFR-1, -2, -3, approved for the treatment of metastatic colorectal cancer (mCRC) and gastrointestinal stromal tumors (17); Axitinib (INLYTA, Pfizer, NY, USA), for the treatment of advanced renal cell carcinoma (18); and Apatinib that hold promise in gastric cancer (19). All these molecules have demonstrated modest efficacy as single agents due to their elevated toxicity, resulting from the inhibition of multiple kinases, leading to limited drug exposure at the maximum tolerated dose. In addition, cumulative evidence raises concerns about other important, although minor, adverse reactions associated with the use of VEGF inhibitors, including aortic dissection (20). Thus, anti-angiogenesis has been incorporated into combination treatments, including standard radiotherapy or chemotherapy, and, most recently, immunotherapy, with variable success depending on the cancer type (21).

Different strategies exist to specifically target cell signaling through VEGFR3/FLT4/CD310 including blocking mAbs, VEGFC trap molecules (22), and small-molecule inhibitors (23). These approaches have significantly reduced tumor lymphatic vessels and metastases in pre-clinical models of cancer. For example, treatment with VEGFR3-blocking antibodies or sVEGFR3 as a decoy receptor reduced chemotherapy-induced lymphatic metastases in models of breast carcinoma (24). Additionally, immunization with antibodies against VEGF-C inhibited the growth of experimental renal and breast carcinomas in mice (22, 25). Unfortunately, although promising in the pre-clinical setting, these antibodies present minimal anti-tumor activity in clinical trials (26).

Specific VEGFR small-molecule inhibitors have been developed with higher affinity for VEGFR3 than for the other family receptors, VEGFR1 and VEGFR2. Among them are MAZ-51, 3-(4-Dimethylamino-naphthalen-1-ylmethylene)-1, 3-dihydroindol-2-one, an indolinone-based synthetic molecule that inhibits the phosphorylation of the tyrosine kinase receptor VEGFR-3 (27), and SAR131675 ((R)-2-amino-1-ethyl-7-(3-hydroxy-4-methoxy-3-methylbut-1-yn-1-yl)-N-methyl-4-oxo-1,4-dihydro-1,8-naphthyridine-3-carboxamide), a potent and selective VEGFR3 inhibitor with an IC_50_ of about 20 nM ten times lower than for the other VEGFR, that significantly reduced tumor size and metastases in mouse models of breast (28) and colon cancer (29). This molecule, although promising in the preclinical setting, was discontinued from further clinical development due to adverse metabolic effects urging the development of less toxic VEGFR3 tyrosine kinase inhibitors such as EVT801 (30). EVT801 is a highly selective VEGFR-3 inhibitor currently in early-phase clinical development (NCT05114668) in patients with advanced or metastatic solid tumors. Its design focuses on modulating lymphangiogenesis and the immunosuppressive tumor microenvironment, particularly by reducing hypoxia and enhancing CD8^+^ T cell infiltration. This molecule represents a promising next-generation approach that is still under clinical scrutiny (30).

In this context, Fruquintinib/FRUZAQLA (6-[6,7-dimethoxyquinazolin-4-yloxy]-N,2-dimethylbenzofuran-3-carbozamide) was developed as a second-generation oral, selective tyrosine kinase competitive inhibitor in 2005. It exhibits a sixty-fold higher affinity (IC50) for VEGFR3/FLT4/CD310 (0.5 nM) compared to VEGFR2 and VEGFR1 (33 nM), with weak inhibition of other tyrosine kinase receptors (31). This higher affinity reduces off-target toxicity, making it possible to achieve higher drug exposure levels with more sustained VEGFR inhibition.

Fruquintinib inhibited the VEGF/VEGFR pathway in *in vitro* assays (32) and in animal models of colon cancer where it showed less toxicity than its predecessors while suppressed angiogenesis and tumorigenesis in combination with doxorubicin and oxaliplatin (33) or anti-PD-1-based immunotherapy.

In clinical trials, FRUZAQLA/ELUNATE has been assessed as monotherapy for the treatment of adult patients with metastatic colorectal cancer (mCRC) who have previously received standard therapies, as well as anti-VEGF and anti-EGFR agents, and have progressed or are intolerant to treatment (34). In the FRESCO-2 trial, fruquintinib significantly improved overall survival and progression-free survival in patients with metastatic colorectal cancer (mCRC) who had exhausted standard treatment options (median overall survival was 7.4 months for the fruquintinib group, compared to 4.8 months for the placebo group) (35).

In 2018, fruquintinib was approved in China for the treatment of patients with metastatic colon cancer refractory to second and third-line treatments (36). In November 2023, fruquintinib received approval from the US FDA for patients with the same indication (37), and in 2024, it was approved in the EU (EMEA/H/C/005979, UK (ID6274), Canada (38), Australia (39), and Switzerland (Marketing authorization n°: 69524) as part of the joint initiative of the Access Consortium. There are currently several open trials that study the efficacy of fruquintinib in solid carcinomas, prominently lung cancer, with varied results (40, 41).

Fruquintinib is generally well-tolerated, and its safety profile is well-documented in several clinical trials. Manageable side effects include hypertension, hand-foot syndrome, fatigue, and gastrointestinal disturbances. Although less frequent, more serious adverse effects such as gastrointestinal perforation and non-specific hemorrhage underscore the need for careful follow-up of patients (42, 43).

In this work, we investigated the impact of fruquintinib, on the myeloid compartment of syngeneic models of murine colorectal and breast cancer. Specifically, we explored its contribution to the modulation of tumor- associated myeloid populations, including macrophage-derived lymphatic endothelial cell progenitors (M-LECP) known to play a determinant role in lymphatic cell proliferation and resistance to chemotherapy. We observe that fruquintinib treatment besides reshaping the tumor immune microenvironment to enhance antitumor immunity, improves therapeutic response to taxane-based chemotherapy on MC38 colorectal murine model.

## 2 Materials and methods

### 2.1 Cell culture

Mouse Colon Carcinoma 38 (MC38), Colon Tumor 26 (CT26), Erlangen 0771 Mammary Carcinoma (E0771), and 4^th^ Tumor 1^st^ Clone (4T1) cell lines were obtained from the American Type Culture Collection (ATCC). RAW 264.7 cells (a murine macrophage-like cell line; ATCC^®^) and immortalized mouse lymphatic endothelial cells (IMLECs), kindly provided by Cornelia Halin (ETH, Zurich), were cultured under standard conditions as described below. Cell lines were routinely tested for mycoplasma contamination using the MycoAlert™ Mycoplasma Detection Kit (Lonza, Switzerland), following the manufacturer’s instructions.

The tumor cell lines MC38 and E0771 cells were cultured in DMEM (Thermo Fisher Scientific, USA), supplemented with 20 mM HEPES and GlutaMAX, and containing 4.5 g/L D-glucose. The cell lines CT26 and 4T1 were cultured in RPMI-1640 (Thermo Fisher Scientific, USA). Both cell media were further supplemented with 10% Fetal bovine serum (FBS) (Teknovas, Spain), 100 U/mL penicillin–100 µg/mL streptomycin (Thermo Fisher Scientific, USA), and 50 µM 2-mercaptoethanol (Thermo Fisher Scientific, USA). All cell cultures were maintained at 37°C in a humidified atmosphere with 5% CO_2_ and 95% relative humidity.

IMLECs are conditionally immortalized cells via the expression of the SV40 large T antigen by culturing them at 33°C in the presence of 50 ng/mL IFN-γ (BioLegend, USA). Cells were maintained in EGM-2 medium (Lonza) supplemented with 5% FBS (Teknovas, Spain) and 1% penicillin–streptomycin (Invitrogen). For differentiation into a primary lymphatic endothelial phenotype, cells were cultured at 35°C in the absence of IFN-γ to inactivate SV40 expression.

### 2.2 Reagents

Fruquintinib and nab-paclitaxel (nab-PTX) were purchased from MedChemExpress (Madrid, Spain). For *in vitro* experiments, fruquintinib and nab-PTX were dissolved in DMSO when preparing stock solutions and further diluted in complete DMEM culture medium for working solutions. For *in vivo* experiments, fruquintinib was administered orally in a volume of 200 µL of 0.6% methylcellulose (MC) and 0.5% Tween 80. Nab-PTX was dissolved in 100 µL of 0.9% NaCl and administered intravenously.

Recombinant murine colony-stimulating factor (M-CSF) was purchased from BioLegend (San Diego, USA). Lipopolysaccharide (LPS) from *Escherichia coli* O111:B4 was purchased from InvivoGen (San Diego, USA).

### 2.3 RNA extraction and real-time qPCR

Total RNA was extracted from cell pellets using the *Monarch^®^ Total RNA Miniprep Kit* (New England Biolabs, Ipswich, USA), following the manufacturer’s protocol. RNA concentration and purity were assessed by spectrophotometry, and samples were stored at -80°C until use.

Reverse transcription was performed using *Omniscript Reverse Transcriptase* (QIAGEN, Hilden, Germany) according to the manufacturer’s instructions. For each reaction, 1–2 μg of total RNA was mixed with 10ng/µl random hexamer primers (Merck-Roche, Mannheim, Germany), 5mM DTT (dithiothreitol; QIAGEN, Hilden, Germany), and 1 mM dNTP Mix (QIAGEN, Hilden, Germany) in a final volume of 10–20 µL. Synthesized cDNA was stored at -20°C until use in quantitative PCR (qPCR).

The detected genes were chosen based on previously published studies (44, 45) and designed according to the coding sequences (CDS) in the NCBI database. All sequences are listed in **Supplementary Table 1**.

Quantitative PCR (qPCR) reactions were carried out using the iQ™ SYBR^®^ Green Supermix (Bio-Rad, Hercules, CA, USA), following the manufacturer’s recommendations. Each 10 µL reaction contained 1 µL of cDNA template (diluted 1:2 to 1:4), 0.5 µM of each primer, and SYBR Green Supermix (1X). Amplification and fluorescence detection were performed on the *CFX Connect Real-Time PCR Detection System* (Bio-Rad, Hercules, CA, USA). Relative expression levels related to untreated cells were calculated using the 2^-ΔΔCt method and normalized to housekeeping gene β-actin. Cycling conditions included an initial denaturation at 95°C for 3 minutes, followed by 44 cycles of 95°C for 15 seconds and 60°C for 20 seconds. To verify the specificity of the amplification products, we performed a melting curve analysis.

### 2.4 Isolation and differentiation of bone marrow-derived macrophages

Bone marrow cells were isolated from the tibia and femur of adult C57BL/6J mice (8–12 weeks old). A total of 2 × 10^6^ cells per well were seeded into 6-well plates pre-coated with 10 μg/mL fibronectin (Sigma-Aldrich). The culture medium was supplemented with 10 ng/mL M-CSF from the first day until the end of the experiment. At day three, 60 ng/mL LPS was added in the absence or presence of 5 μM fruquintinib for an additional 72 hours. On Day 6, cells were collected for analysis.

### 2.5 IMLECs microtubule formation essay

Flat-bottom 96-well plates were pre-coated with 70 μL per well of basement membrane matrix (Corning Matrigel^®^, Cat. No. 354234; BD Biosciences). Plates were then incubated at 37°C for 30 minutes to allow gel polymerization. Primary IMLEC cells were seeded onto precoated wells at a density of 2 × 10^4^ cells per well in complete EGM-2 medium. Cells were incubated for 18 hours in the presence or absence of fruquintinib (0.03 μM, 0.3 μM, and 3 μM) at 37°C in a humidified atmosphere containing 5% CO_2_. The concentrations selected were similar to the ones reported to inhibit tubule formation in human endothelial cells (32). Images were captured using a Nikon SMZ18 stereo microscope. Tube formation was quantified using the Angiogenesis Analyzer plugin for ImageJ (NIH, USA), measuring parameters including the number of isolated segments and the number of segments.

### 2.6 Flow cytometry analysis

Tumor tissues were enzymatically and mechanically digested using collagenase D (400U/mL; Roche Diagnostics, Mannheim, Germany) and DNase I (50 µg/mL; Roche Diagnostics, Mannheim, Germany) at 37°C for 15 minutes. Red blood cells were lysed in ACK (ammonium-chloride-potassium) buffer. When collected, the tumor-draining lymph nodes (LNs) were mechanically dissociated and filtered through a 0.4 μM mesh to obtain single-cell suspensions.

Cell viability was determined by staining with the viability dye Zombie NIR™ (BioLegend, San Diego, CA, USA) for 10 minutes in the dark at room temperature. Afterwards, cells were stained with fluorochrome-conjugated antibody mixtures for 15 minutes at 4°C. A complete list of antibodies used is provided in **Supplementary Table 2**.

Samples were acquired using a CytoFLEX S flow cytometer (Beckman Coulter, USA), and the data were analyzed with FlowJo software, version 10 (BD Biosciences, USA). Gating strategies are provided in **Supplementary Figures 1-5**. To account for variability in tumor size between treatment groups, immune cell populations were expressed as percentages of CD45^+^ leukocytes rather than absolute counts per tumor. Absolute numbers are strongly influenced by tumor mass and total cellularity, which can confound interpretation when comparing treated and untreated groups. Relative representation of subpopulations (e.g., CD8^+^ T cells, Tregs, macrophage subsets) provides a more accurate measure of changes in immune composition within the tumor microenvironment. This approach is consistent with recommendations from the International Immuno-Oncology Biomarker Working Group and widely adopted practices in immuno-oncology studies (46–49).

### 2.7 Syngeneic heterotopic mouse cancer models and therapeutic schedules

Animal procedures were conducted following protocols approved by the Animal Ethics Committee of the University of Navarra (Protocol No. 114–21) and complied with institutional and European guidelines for animal care and use (Directive 2010/63/EU).

Female and male BALB/c and C57BL/6J mice (6–7 weeks old) were obtained from ENVIGO (Huntingdon, UK). Transgenic Kikume Green-Red (KikGR) mice [B6.Cg-Tg(CAG-KikGR)1Ymag] were purchased from The Jackson Laboratory. All animals were housed in the institutional animal facility under standard conditions.

For tumor model generation, mice were anesthetized by intraperitoneal injection of a ketamine/xylazine mixture. The anesthetic solution was prepared by mixing 450 µl of sterile saline solution, 450 µl of ketamine hydrochloride (Ketamidor 100 mg/ml, injectable solution, Karizoo Richter-Pharma, Barcelona, Spain), and 100 µl of xylazine (Rompún 20 mg/ml, Bayer, Leverkusen, Germany) were combined to obtain final concentrations of 45 mg/ml ketamine and 2 mg/ml xylazine. Each mouse (~20 g) received 40 µl of this mixture, corresponding to doses of approximately 90 mg/kg ketamine and 4 mg/kg xylazine.

Tumor cells were subcutaneously implanted in C57BL/6J (MC38 and E0771 cells) or BALB/c (CT26 and 4T1 cells) mice strains. Tumor cells were injected at different concentrations depending on their growth rate: 7 × 10^5^ cells for MC38, CT26, and E0771 cells, and 5 × 10^5^ cells for breast carcinoma 4T1 cells. Fruquintinib was administered orally at 5 mg/kg (unless otherwise indicated), starting when tumors were palpable (approximately 50 mm³). For combination experiments, nab-PTX was administered intravenously at a dose of 10 mg/kg every two to three days, for a total of five doses.

Tumor growth was monitored regularly using a caliper, and tumor volume was calculated as (length × width²)/2. Mice were humanely euthanized by CO_2_ inhalation, and tumors, lungs, lymph nodes, or bone marrow were harvested and processed for flow cytometry or immunohistochemistry (IHC) analysis, according to each experiment.

To study survival in an advanced-stage, CT26 colon carcinoma cells were subcutaneously inoculated in BALB/c mice. When tumors reached an average volume of approximately 600 mm³, fruquintinib was administered daily at a dose of 5 mg/kg in a volume of 200 µL for 21 consecutive days. When tumors reached the endpoint size (18 × 18 mm) or animals exhibited signs of distress (e.g., lethargy, severe discomfort), they were humanely euthanized.

Mice were euthanized by gradual exposure to carbon dioxide (CO_2_), following the recommendations of the AVMA Guidelines for the Euthanasia of Animals (2020). Compressed CO_2_ was introduced into the chamber at a flow rate equivalent to 20% of the chamber volume per minute (approximately 1.5–2.0 L/min for a standard mouse cage), until respiration ceased. The gas flow was maintained for at least 1 minute after apparent respiratory arrest to ensure death. Death was confirmed by the absence of heartbeat and reflexes prior to disposal.

### 2.8 Immunohistochemistry

For immunohistochemistry, tumors were harvested, fixed in formalin, and embedded in paraffin. Sections of 3 μm were deparaffinized through consecutive alcohol solutions. Antigen retrieval was performed using the PT-Link system (Agilent, Santa Clara, USA) in RT-Link buffer ARS6 (Akoya Biosciences, Marlborough, USA). Sections were incubated overnight with the primary anti-CD31 antibody (Hystosure, Nottingham, UK) diluted 1:200 in Protein Block or anti-EpCAM antibody diluted 1:1000 in Protein Block (Abcam, Cambridge, UK) at 4°C. Afterwards, they were incubated in horse rabbit peroxidase conjugated goat anti-rat secondary antibody for 30 minutes at room temperature. The complete list of antibodies used and experimental conditions is provided in **Supplementary Table 3**. Signal detection was carried out using a DAB substrate (1:1000 dilution) for the detection of CD31+ vessels and was monitored under a light microscope. Slides were counterstained with hematoxylin (Leica Biosystems, Wetzlar, Germany) for 1 minute, and mounted using VITROCLUD^®^ mounting medium for microscopy (Deltalab, Spain).

Digital images were acquired using the Aperio CS2 scanner (Leica Biosystems), and image analysis was performed with QuPath software (version 0.5.1). For the manual count of intratumoral CD31+ vessels, five regions of 500 μm × 500 μm were selected from each histological tumor sample and evenly distributed across the entire tumor section. The number of vessels in each area was then counted. For detection of EpCAM-positive lung metastatic tumor areas in 4T1 breast adenocarcinoma mice, individual macrometastatic nodules on each mouse were manually counted.

### 2.9 FLT4/VEGFR3 expression and prognostic analysis using public databases

The FLT4 (VEGFR3/CD310) gene expression analysis was performed using TNMplot (50), which integrates RNA-Seq data from GTEx, TCGA, and TARGET databases to compare normal, tumor, and metastatic samples (51). Additionally, subgroup-specific and survival analyses for colorectal cancer samples were performed using UALCAN (52), a platform designed to facilitate the visualization of tumor-specific gene expression and prognosis assessments based on TCGA RNA-Seq data (53, 54).

### 2.10 Statistical analysis

All statistical analyses were performed using GraphPad Prism software (version 9.5.0). For comparisons between two groups, Student’s t-test was used when data met the assumptions of normality and homoscedasticity; otherwise, the non-parametric Mann-Whitney U test was applied. For multiple group comparisons relative to untreated or vehicle control groups, one-way analysis of variance (ANOVA) followed by appropriate *post hoc* tests were used; if parametric assumptions were not met, the Kruscal-Wallis test with corresponding *post hoc* analyses was employed. Survival curves were estimated using the Kaplan-Meyer Method, and differences in survival were assessed by the long-rank (Mantel-Cox) test. Statistical significance was defined as p value of <0.05. When differences were statistically significant (* for a *p* value <.05, ** for a *p* value <.01, *** for a *p* value <.001, **** for a *p* value <.0001). Error bars indicate the standard error of the mean (SEM).

## 3 Results

### 3.1 Fruquintinib inhibits tumor growth, reduces metastatic burden, and improves survival across multiple syngeneic tumor models

We started our experimentation by confirming the antitumor efficacy of fruquintinib in colon and breast carcinoma models in our experimental set up. To this aim, we employed four well-established syngeneic tumor models: MC38 and CT26, both murine colorectal carcinoma cell lines, and E0771 and 4T1, representing murine breast cancer. fruquintinib oral treatment (5 mg/kg) was initiated once tumors reached a volume of 50–80 mm³ and continued daily for 15 days, except in the E0771 model, where treatment was administered for 21 days at a dose of 10 mg/kg.

**Figure 1:**
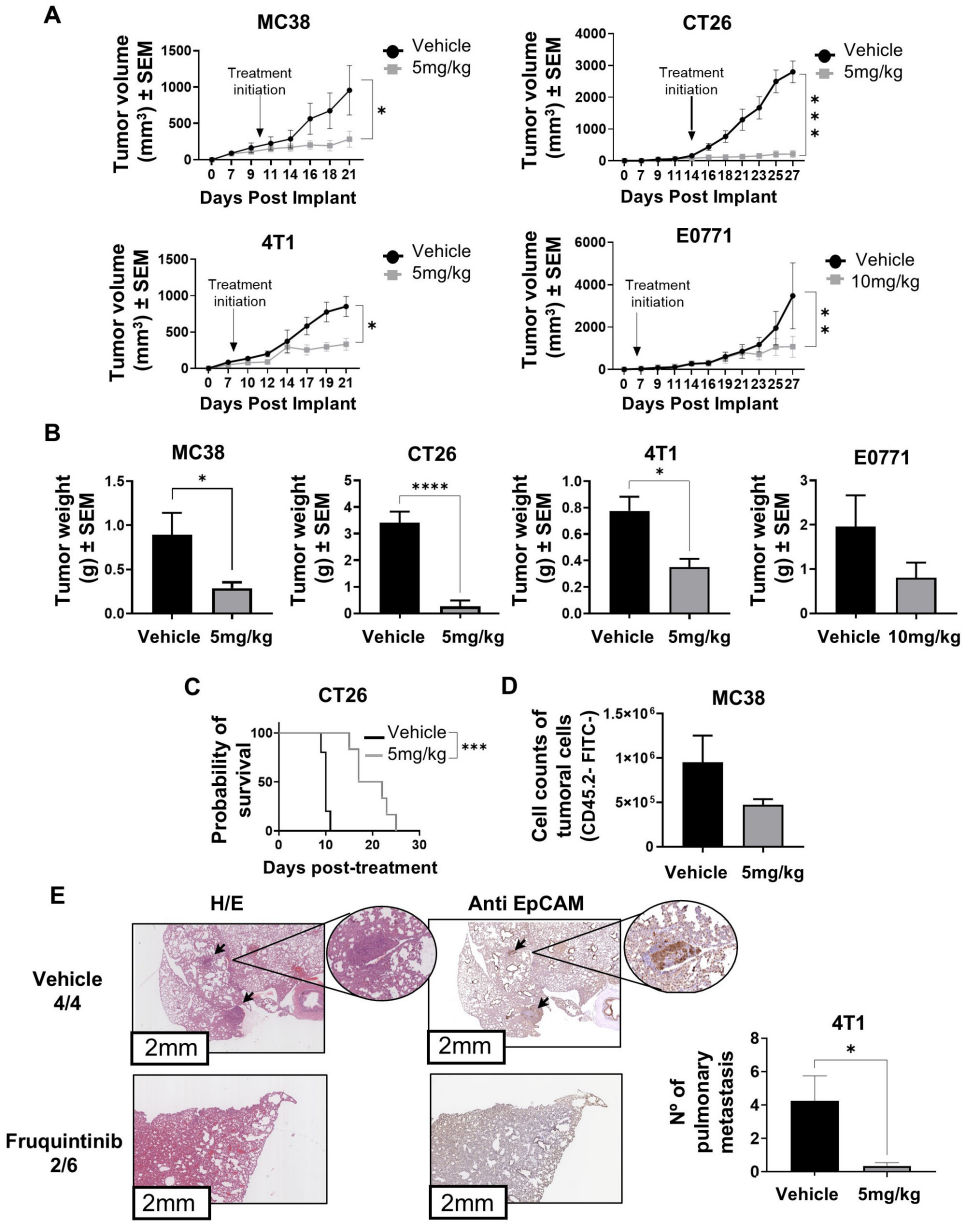
Fruquintinib suppresses tumor progression and metastasis across multiple murine tumor models. **(A)** Tumor volume progression during daily treatment with fruquintinib in the colon: MC38 and CT26, and breast: E0771, and 4T1 mice tumor models. Fruquintinib was administered at a dose of 5 mg/kg in MC38 (*n* = 5 control, *n* = 7 fruquintinib), CT26 (*n* = 5 control, *n* = 7 fruquintinib) and 4T1 (*n* = 4 each group) mouse models and at dose of 10 mg/kg in the case of E0771 tumors once daily for 15 days starting when tumors reached approximately 80 mm³ for MC38, CT26, and 4T1. Mice bearing E0771 tumors received fruquintinib at 10 mg/kg (*n* = 4 control, *n* = 5 fruquintinib) daily for 21 days when tumors reached approximately 50 mm³. Arrows indicate treatment initiation time points for each model. **(B)** Tumor weight at endpoint for each treatment condition on panel **(A, C)** Kaplan–Meier survival analysis in BALB/c mice bearing advanced CT26 tumors. Fruquintinib (5 mg/kg) (*n* = 5 control, *n* = 6 fruquintinib). **(D)** Flow cytometry quantification of tumor cell seeding in the lymph nodes of Kikume transgenic mice bearing MC38 (CD45.2- KikGR-) tumors, and treated or not with fruquintinib (5 mg/kg daily) for 14 consecutive days. (*n* = 5 control, *n* = 6 fruquintinib). **(E)** Quantification of macrometastatic lung nodules in 4T1-bearing mice after 19 days of fruquintinib treatment (5 mg/kg). Representative images of hematoxylin/eosin (H/E) staining (left) and immunohistochemistry using a specific anti-EpCAM antibody (right). The fraction of mice with detectable metastases is indicated as the number of mice with metastases over the total number of mice in each group. The adjacent graph shows the quantification of metastatic nodules per mouse in control and fruquintinib-treated groups. Digital images were acquired with an Aperio CS2 scanner (Leica Biosystems), and macrometastases were manually counted using QuPath software (version 0.5.1). Group sizes were n = 4 for control and n = 6 for fruquintinib-treated mice. Data are presented as mean ± SEM. Statistical analyses were performed using an unpaired t-test for tumor volume and weight and metastasis counts, and the log-rank (Mantel–Cox) test for survival. *p < 0.05 **p < 0.01 ***p < 0.001 and ****p < 0.0001. Data represent one independent experiment of each *in vivo* experiment. *n*: number of mice per group.

As shown in **Figure 1A**, fruquintinib treatment resulted in significant inhibition of tumor growth across all tumor models tested. Notably, tumor growth suppression was evident in the CT26 (*p* < 0.001), MC38 (*p* < 0.05), and 4T1 (*p* < 0.05) models at a dose of 5 mg/kg. In contrast, the E0771 model required a higher dose (10 mg/kg) and a more extended treatment duration (21 days) to achieve comparable effects (*p* < 0.01). Final tumor weights were consistent with these observations, showing significant reductions in all treated groups compared to controls, except in the E0771 model (**Figure 1B**).

We next tested whether fruquintinib could be effective in a model that simulated a late-stage colon cancer by injecting CT26 cells. In this case, treatment was initiated at a tumor volume of approximately 600 mm³. We observed that fruquintinib was also able to significantly prolong overall survival (*p* < 0.001), as demonstrated by Kaplan–Meier analysis (**Figure 1C**). In addition, we wanted to investigate the impact of fruquintinib on metastasis seeding to draining lymph nodes (dLNs). To this end, we implanted MC38 cells into Kikume transgenic mice that constitutively express Kik green fluorescent protein, enabling the identification of tumor cells against a non-fluorescent background. Mice received daily treatment with fruquintinib (5 mg/kg), and on day 14, the dLNs were harvested for flow cytometric analysis of KikGR^+^ (endogenous) and KikGR- (tumor) cells to detect tumoral metastatic burden. As observed in **Figure 1D**, fruquintinib reduced the presence of tumor cells in the draining lymph nodes. We continue by analyzing its efficacy to block metastases in this case, using the highly metastatic 4T1 breast carcinoma cells. As shown in **Figure 1E**, daily treatment with fruquintinib significantly decreased pulmonary metastases in the 4T1 mouse model. Histological analysis by hematoxylin and eosin (H/E) staining revealed the presence of visible metastatic nodules in the lungs of all mice in the vehicle-treated group (4/4), whereas only 2 out of 6 fruquintinib-treated mice developed lung metastases. Consistently, immunohistochemical staining for EpCAM, a marker of epithelial tumor cells, confirmed these findings, showing markedly reduced tumor cell colonization in the lungs of fruquintinib-treated animals compared to controls. Quantitative analysis demonstrated a significant reduction in the number of pulmonary metastatic nodules per mouse, decreasing from an average of 3.5 ± 1.6 nodules in the vehicle group to 0.33 ± 0.21 nodules in the fruquintinib-treated group (p < 0.05) (**Figure 1E**). These results indicate that fruquintinib effectively suppresses lung metastasis formation in this aggressive breast cancer model.

Together, these results demonstrate that fruquintinib exerts broad antitumor activity across multiple breast and colon mouse syngeneic models, effectively inhibiting primary tumor growth, limiting metastatic spread, and extending survival in advanced disease settings.

### 3.2 Fruquintinib inhibits vascularization *in vivo* and *in vitro*

**Figure 2:**
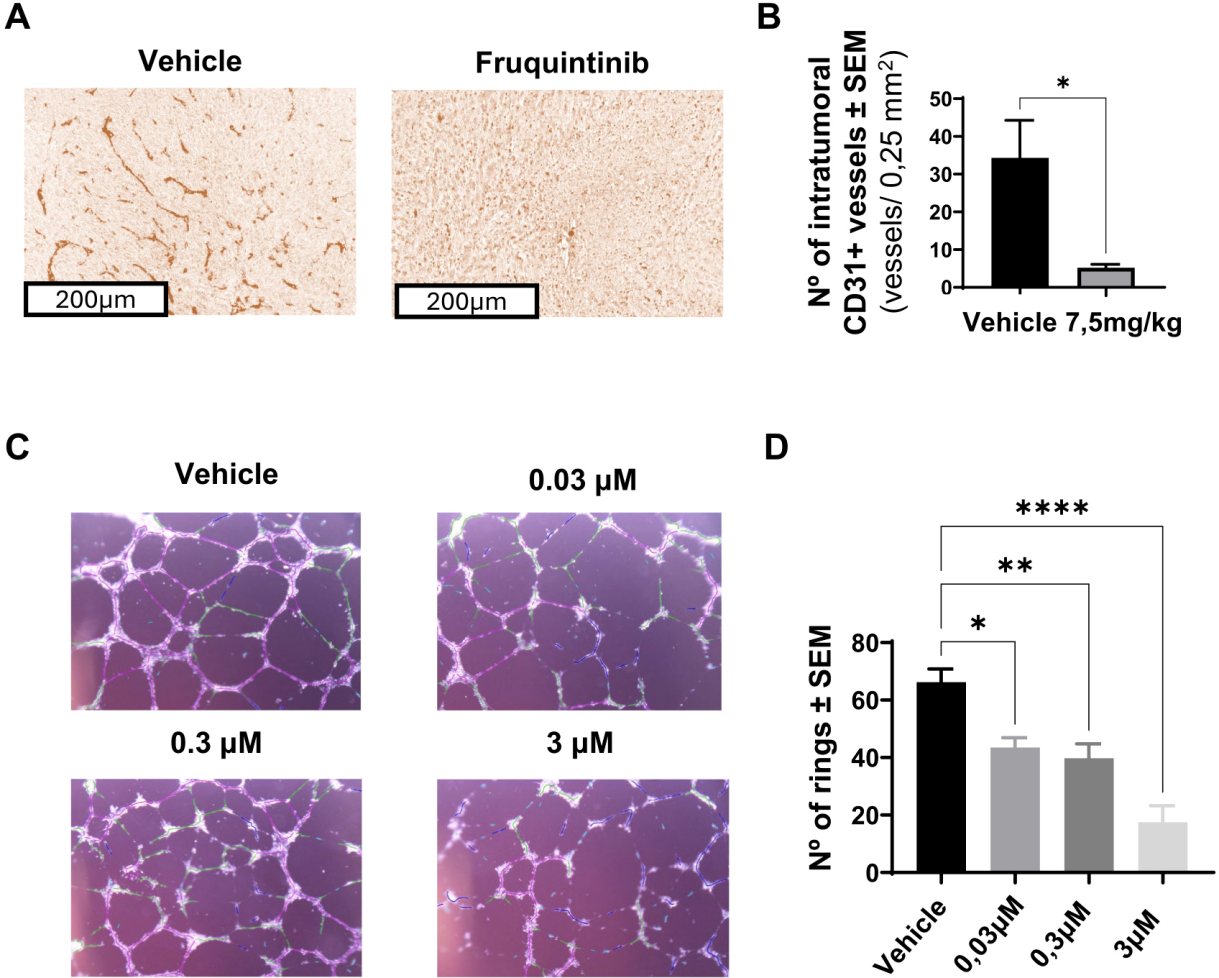
Fruquintinib inhibits tumor vascularization and impairs *in vitro* IMLEC tube formation. **(A)** Representative image showing immunohistochemical staining for CD31 in tumor sections from MC38 tumors treated or not with fruquintinib (7,5 mg/kg) for 19days. **(B)** Graph showing absolute numbers of CD31-positive vessels per field. *n* = 4 mice on control group, *n* = 6 mice on fruquintinib group on one independent experiment. **(C)** Representative microphotograph showing IMLEC tube formation on assay on matrigel after 18 h of incubation with increasing concentrations of fruquintinib (0.03, 0.3, and 3 µM). **(D)** Graph showing the mean number of endothelial rings per condition (one independent experiment of *n* = 4 per condition). Data are presented as mean ± SEM. Statistical significance was determined using an unpaired t-test for CD31^+^ immunohistochemistry, and one-way ANOVA with multiple comparisons for the IMLEC tube formation assay. *p < 0.05 **p < 0.01 and ****p < 0.0001.

As fruquintinib activity targets VEGFR receptors, we next examined the impact of fruquintinib on tumor-associated vasculogenesis. To this aim, we analyzed the presence of blood (CD31^+^) vessels in tumors extracted from MC38-bearing mice treated for 19 days with fruquintinib (7.5 mg/kg). Analyses of CD31-positive vessels by immunohistochemistry revealed a significant decrease in CD31-positive vessels per 0,25 mm² in the treated group when compared to controls (from 34.3 ± 9.95 to 5.2 ± 0.89 CD31^+^ vessels per area), indicating a marked decrease in tumor vascularization (*p* < 0.05, **Figures 2A, B**). Gross inspection of tumors also showed an evident reduction in peritumoral blood vasculature in fruquintinib-treated tumors compared to controls (**Supplementary Figure 6**).

Complementary *in vitro* studies using immortalized mouse lymphatic endothelial cells (IMLECs) cultured on matrigel substrates demonstrated that fruquintinib impaired lymphatic endothelial tube formation in a dose-dependent manner (**Figures 2C, D**). Additionally, the anti-proliferative effect of fruquintinib on SVEC cells was confirmed by measuring the incorporation of the vital dye neutral red, while its anti-migratory effect was assessed through wound-healing assays performed on SVEC monolayers (**Supplementary Figure 7**). In the present manuscript, we demonstrate that fruquintinib inhibits tubule formation in mouse lymphatic endothelial cells (IMLECs) (**Figure 2C**) at concentrations comparable to those that inhibit tubule formation in HUVECs (32). These findings suggest that the potency of fruquintinib against murine VEGFRs is very similar to that observed in humans, with minimal differences likely attributable to pharmacokinetic factors rather than binding affinity. This comparable affinity may stem from the conserved structure of VEGFR3 between mouse and human, with high sequence conservation (~90%) in the kinase domains (55), suggesting that fruquintinib’s binding affinity and inhibitory potency are conserved across these species.

In summary, these findings confirm that fruquintinib exerts potent inhibitory effects on the tumor vasculature in the MC38 colon carcinoma model, a key mechanism for limiting tumor growth and metastatic progression.

### 3.3 Fruquintinib alters myeloid-cell populations in the tumor and the bone marrow

VEGFR3/FLT4/CD310 receptor is known to be expressed in stromal cells that contribute to tumor growth and survival, and the use of VEGFR3 inhibitors affects tumor infiltrate. To ascertain whether the immune population could be a direct target of VEGFR3/FLT4/CD310 inhibition, we first evaluated CD310 expression in tumor immune cells and in the bone marrow of mice bearing and non-bearing tumors.

**Figure 3:**
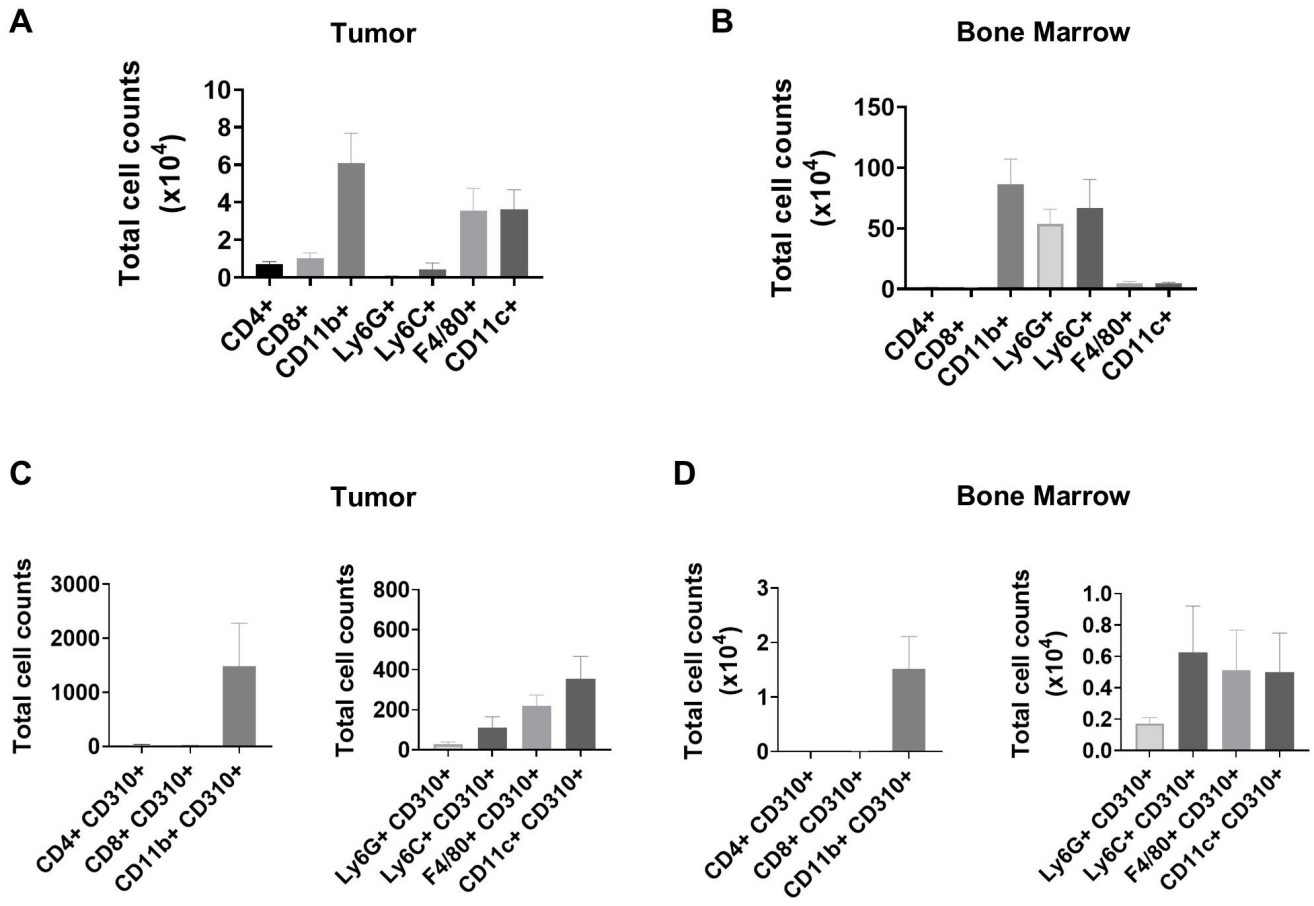
VEGFR3/FLT4 is expressed in myeloid cells of tumors and the bone marrow. Cell counts from gated viable leukocytes (CD45^+^) of CD4^+^, CD8^+^ T lymphocytes, CD11b^+^ myeloid cells, CD11b^+^ Ly6C^+^ monocytes, CD11b^+^ F4/80^+^ macrophages and CD11b^+^ CD11c^+^dendritic cells on tumor **(A)** and bone marrow **(B)** of MC38 tumor-bearing mice. Cell counts of CD310^+^ (VEGFR3/FLT4) cells in the same immune populations as in A and B in the tumor **(C)** and in the bone marrow of tumor-bearing mice **(D)**. Bone marrow data were obtained from one-tenth of the total marrow cell suspension. Data represent mean ± SEM of two independent experiments with *n* = 6 mice per group.

General cell counts of immune population within tumor-resident viable leukocytes revealed that F4/80^+^ macrophages and CD11c^+^ dendritic cells were the most abundant, followed by CD4^+^ and CD8^+^ lymphocytes, CD11b^+^ Ly6C^+^ monocytes and CD11b^+^ Ly6G^+^neutrophils (**Figure 3A**). In bone marrow, CD11b^+^ myeloid cells represented the major immune compartment, principally composed of Ly6G^+^ neutrophils and Ly6C^+^ monocytes, along with minor fractions of CD11c^+^ dendritic cells and F4/80^+^ macrophages (**Figure 3B**).

In the case of CD310^+^ expression, in tumor samples, cell counts from alive leukocytes, showed that myeloid CD11b^+^ cells constitute the most abundant immune population expressing CD310^+^ within the tumor microenvironment, markedly exceeding the numbers of CD4^+^ CD310^+^ and CD8^+^ CD310^+^ T lymphocytes which were practically undetected (**Figure 3C**, left). Analysis of additional cell counts from alive leukocytes of myeloid subsets demonstrated that CD11c^+^ CD310^+^dendritic cells were the most abundant cells followed by F4/80^+^ CD310^+^macrophages and Ly6C^+^ CD310^+^monocytes whereas only very few Ly6G^+^ CD310^+^ cells were detected (**Figure 3C**, right).

In the bone marrow of MC38 tumor-bearing mice, CD310^+^ was expressed mostly on CD11b^+^myeloid cells but was absent from T CD8^+^and T CD4^+^ lymphocytes. In this case the counts of Ly6C^+^ CD310^+^monocytes, CD11c^+^ CD310^+^dendritic cells and F4/80^+^ CD310^+^macrophages were similar (**Figure 3D**, left and right). Importantly, bone marrow samples from non-tumor bearing mice exhibited comparable profiles (**Supplementary Figure 8**). Collectively, these data demonstrate a predominant expression of CD310 within myeloid CD11b^+^ cells as opposed to lymphocytes in both MC38 tumors and bone marrow of tumor-bearing mice. Furthermore, although CD11c^+^ dendritic cells represent a minor population in bone marrow, CD11c^+^ CD310^+^ dendritic cells along with Ly6C^+^ CD310^+^ monocytes comprise the largest CD310^+^ myeloid subset.

**Figure 4:**
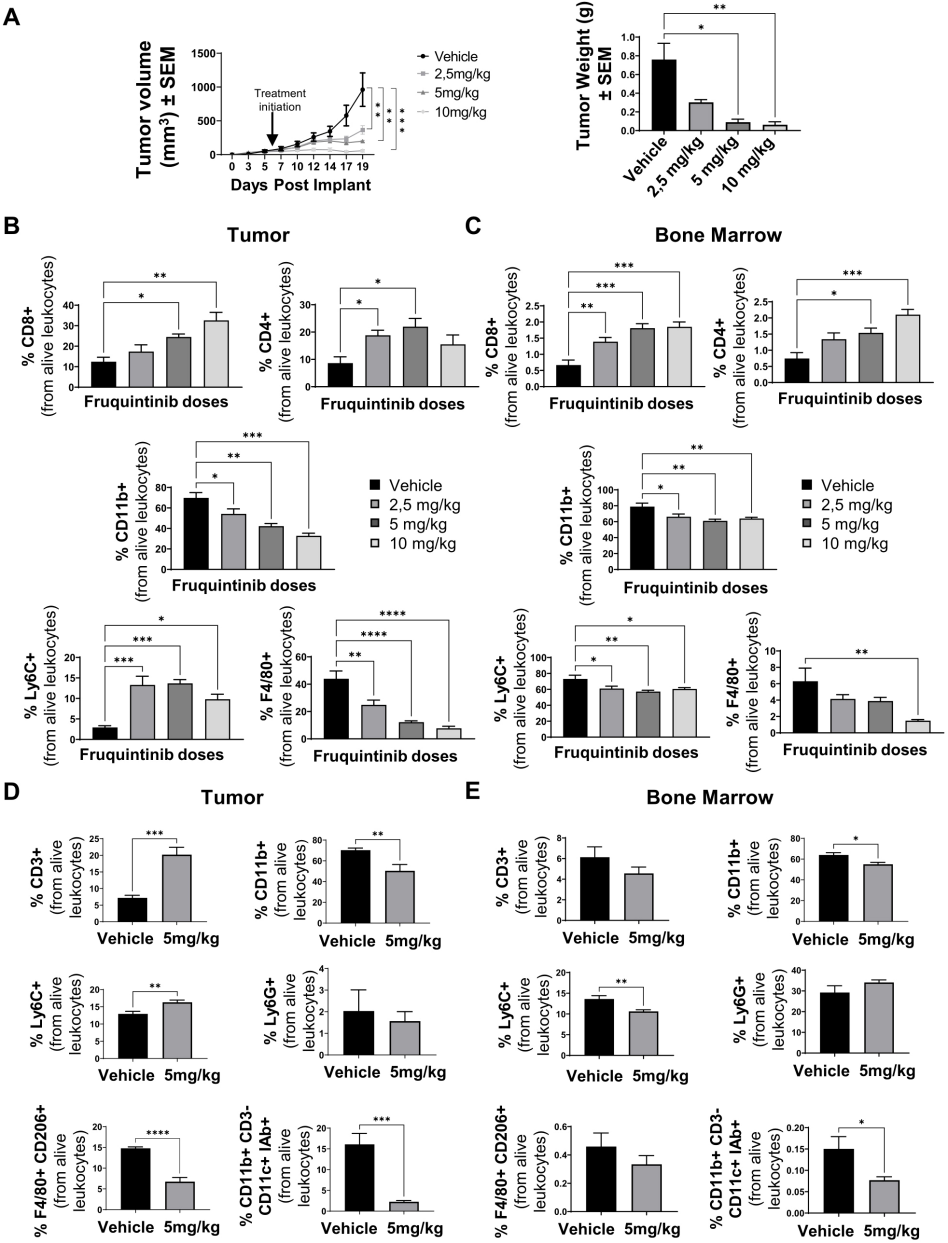
Fruquintinib treatment affects the myeloid immune compartment. **(A)** Evolution of tumor volume (left) and tumor weight (right) of MC38 tumor-bearing mice treated daily with vehicle or fruquintinib at 2.5, 5, or 10 mg/kg, starting when tumors reached approximately 80 mm³ (*n* = 5 per group in one independent experiment). **(B-C)** Flow cytometry quantification of the percentages of CD4^+^ and CD8^+^ T lymphocytes, inflammatory monocytes (Ly6C^+^), macrophages (F4/80^+^), and total myeloid CD11b cells in tumors **(B)** and bone marrow **(C)** related to CD45+ cells in mice treated as in **(A)**. **(D-E)** Flow cytometry quantification of total T lymphocytes (CD3^+^), myeloid cells (CD11b^+^), inflammatory monocytes (Ly6C^+^), neutrophils (Ly6G^+^), M2-like tumor-associated macrophages (CD3^-^CD11b^+^F4/80^+^CD206^+^) and type 2 conventional dendritic cells (cDC2; CD3^-^CD11b^+^CD11c^+^I-A/I-E^+^) **(D)** and in the bone marrow **(E)** of mice bearing MC38 tumors and treated for 17 days at 5 mg/kg fruquintinib (*n* = 6 per group in one independent experiment). Data are presented as mean ± SEM. Statistical analyses were performed using one-way ANOVA or unpaired t-tests as appropriate. *p < 0.05 **p < 0.01 ***p < 0.001 and ****p < 0.0001.

We next investigated whether treatment with fruquintinib would alter the proportion of VEGFR3-expressing immune populations in MC38 tumors and the bone marrow, thereby contributing to curbing the tumor’s immune escape. We selected the murine colon cancer cell line MC38 because fruquintinib has been extensively studied, developed, and clinically approved for the treatment of colorectal cancer in humans (35, 37). Therefore, using a colon-derived cell line is appropriate and consistent with the drug’s therapeutic indication, allowing for a more accurate translational interpretation of the results. To achieve this, MC38 tumor-bearing mice were treated for 13 days with increasing doses of fruquintinib of 2.5, 5, and 10 mg/kg, beginning when tumors were palpable. As expected, fruquintinib treatment reduced tumor weight and volume in a dose-dependent manner (**Figure 4A**), with one out of five mice achieving a complete response at 5 and 10 mg/kg.

Immune profiling revealed a significant, dose-dependent modulation of both lymphoid and myeloid compartments. CD8^+^ and CD4^+^ T cells increased in tumors, while CD11b^+^ myeloid cells decreased. Within the myeloid compartment, CD11b+Ly6C+ immature monocytes increased, whereas CD11b+F4/80+ macrophages decreased significantly, especially at higher doses (p<0.0001) (**Figure 4B**).

In bone marrow, total myeloid cells (CD11b+) and CD11b+Ly6C+ immature monocytes decreased moderately, and CD11b+F4/80+ macrophages were significantly reduced (**Figure 4C**). These results suggest fruquintinib affects the maturation of myeloid cells from Ly6C+ precursors.

To analyze suppression of myeloid subpopulations, mice were treated with fruquintinib (5 mg/kg) for 17 days. Flow cytometry analysis revealed a global decrease in type 2 dendritic cells (cDC2: CD3-CD11b^+^CD11c^+^IAd^+^) and M2-like macrophages (CD3^-^CD11b^+^F4/80^+^CD206^+^) in tumors, as well as an increase in immature monocytes (CD3^-^CD11b^+^Ly6C^+^) in tumors and a decrease in the bone marrow of treated animals. In contrast, the neutrophil (CD3^-^CD11b^+^Ly6G^+^) population remained unaffected in both organs analyzed (**Figures 4D, E**). We also confirmed by immunohistochemistry the increase in Ly6C^+^ cells and by immunofluorescence the decrease of CD11b^+^ myeloid cells and F4/80^+^ macrophages in MC38 tumor samples following fruquintinib treatment in another independent experiment (**Supplementary Figures 9, 10**). Similarly, myeloid immune modulation was also observed in the 4T1 and CT26 tumor models, with a decrease in the CD11b+ myeloid subsets, specifically in F4/80+ macrophages and cDC2 cells (**Supplementary Figure 11**).

Thus, treatment with fruquintinib may alter the myeloid-monocytic differentiation in the tumor and in the bone marrow, resulting in a lower amount of myeloid suppressor cells in treated animals.

### 3.4 Fruquintinib suppresses the differentiation of macrophage-derived lymphatic endothelial cell precursors *in vivo* and *in vitro*

Recently, Sophía Ran and collaborators (7) described the existence of macrophage-derived lymphatic endothelial cell progenitors (M-LECP) characterized by concomitant expression of markers of myeloid cells (CD11b^+^), myeloid precursors (Ly6C^+^ Sca1^+^), macrophage (F4/80 or CD206), and lymphatic endothelium (PDPN^+^, LYVE or VEGFR3). This M-LECP population constitutes a macrophage subtype that bridges both VEGFR3-mediated pro-angiogenic and immune-suppressive mechanisms, constituting an interesting target for tumor treatment.

**Figure 5:**
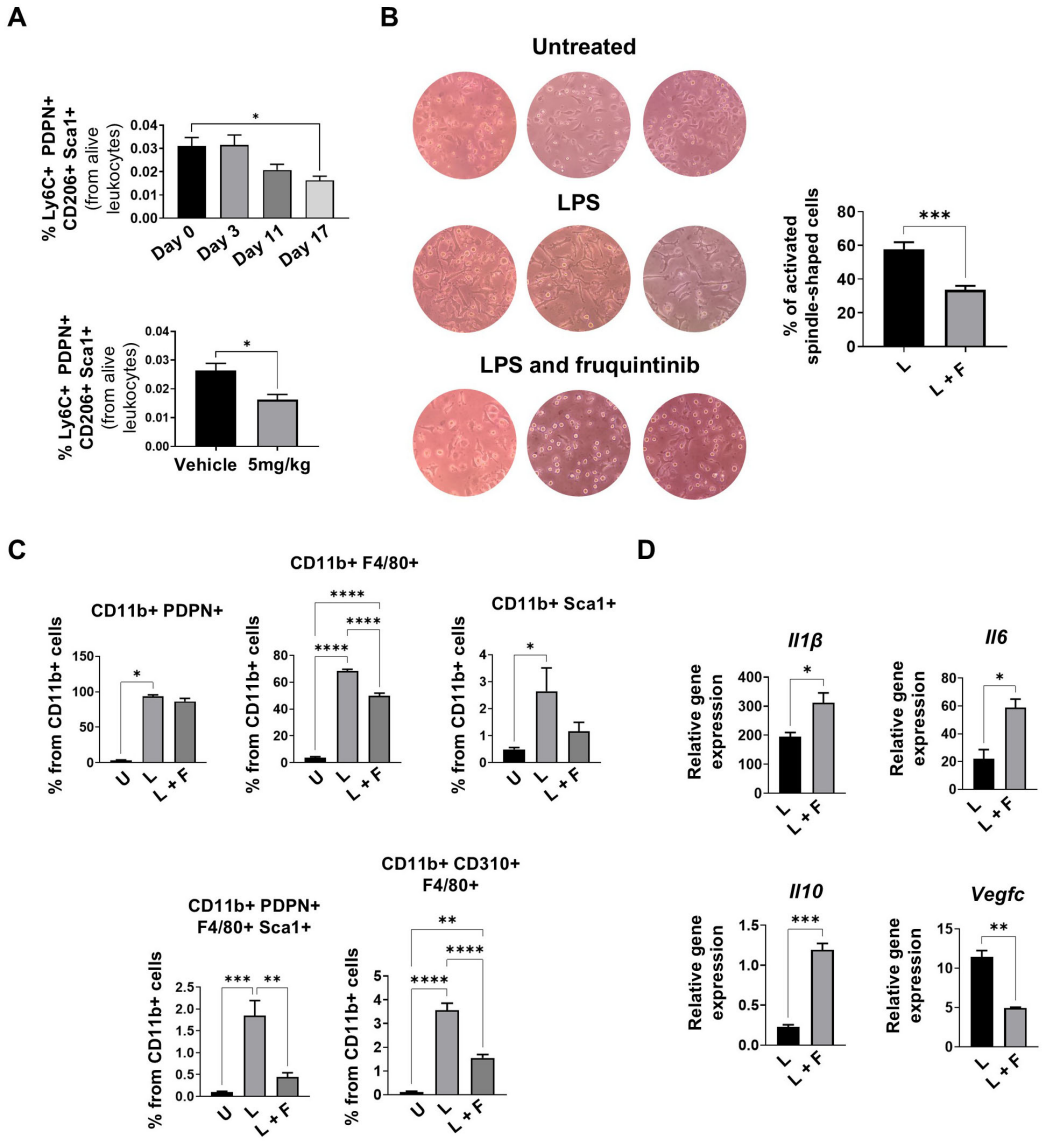
Fruquintinib inhibits *in vitro* M-LECP differentiation. **(A)** Time-course analysis by flow cytometry of M-LECP (CD11b^+^ Ly6C^+^ PDPN^+^CD206^+^ Sca1^+^) frequencies in bone marrow of MC38 tumor-bearing mice treated daily with fruquintinib (5 mg/kg) (one independent experiment of *n* = 6 mice per group). **(B)** Left: representative brightfield microphotographs of bone marrow-derived cells differentiated to M-LECP in the presence or absence of fruquintinib (three independent experiments of *n* = 4 per group). Right quantification of the percentages of cells with spindle morphology. **(C)** Quantification by flow cytometry of podoplanin-positive myeloid cells (CD11b^+^ PDPN^+^), macrophages (CD11b^+^ F4/80^+^), CD11b stem cells (CD11b^+^Sca1^+^), M-LECP (CD11b^+^ PDPN^+^ F4/80^+^ Sca1^+^) and VEGFR3/CD310-expressing macrophages (CD11b^+^ CD310^+^, F4/80^+^), in *ex vivo* differentiated M-LECP populations, in the presence or absence of fruquintinib (5 µM), (two independent experiments of n*=*4 mice per condition). **(D)** Quantification by qPCR of the expression of anti-inflammatory *Il10*, proinflammatory *Il6* and *Il1β* genes and VEGFR3 ligand, *Vegfc*, in the myeloid precursors differentiated in B (two independent experiments of *n* = 3 mice per condition). Data are presented as mean ± SEM. Statistical analyses were performed using one-way ANOVA or unpaired t-tests as appropriate. *p < 0.05, p < 0.01, *p* < 0.001, and ***p < 0.0001. Abbreviations: U, untreated; L, LPS; L+F, LPS + fruquintinib.

To study whether fruquintinib could modulate the presence of M-LECP, we also quantified by flow cytometry the percentages of (CD11b^+^ Ly6C^+^ PDPN^+^ CD206^+^ Sca1^+^) M-LECP in the bone marrow of the mice harboring MC38 tumors and treated for 17 days with fruquintinib at a dosage of 5mg/kg. As shown in **Figure 5A**, M-LECP percentages diminished over time in a dose-dependent manner, being significantly lower at day 17 when compared to day 0 of treatment.

To study whether VEGFR3/FLT4/CD310 inhibition alters the differentiation of M-LECP from its precursor, we *in vitro* model the differentiation of M-LECP from bone marrow precursors by exposing them to M-CSF (10 ng/mL) and LPS (60 ng/mL), in the presence or absence of 5 µM fruquintinib. LPS was used to activate Toll-like receptor 4 (TLR4) signaling as previously described to induce VEGFR3-mediated M-LECP differentiation (44, 45). Representative brightfield images shown in **Figure 5B** demonstrated an attenuation of the typical macrophage morphology when the differentiation was performed in the presence of fruquintinib (*p <* 0.001). Flow cytometric quantification of M-LECP markers revealed, as expected, that LPS markedly increased the frequency of CD11b^+^ cells expressing the canonical M-LECP activation markers PDPN, F4/80, and Sca1. Of interest, fruquintinib treatment led to a significant diminution in the proportions of immature myeloid cells (CD11b^+^Sca1^+^), VEGFR3/FLT4/CD310 expressing macrophages (CD11b^+^F4/80^+^CD310^+^), and M-LECP (CD11b^+^PDPN^+^F4/80^+^ and Sca1^+^) (**Figure 5C**). Additionally, immunohistochemical analysis revealed a significant accumulation of LYVE-1-positive cells in MC38 and 4T1 tumors following fruquintinib treatment. This accumulation may reflect a blockade of M-LECP differentiation, preventing their integration into pre-existing lymphatic vessels and limiting the expression of other typical M-LECP markers (such as F4/80+ or Sca1+), as previously observed in our ex vivo differentiation assays (**Supplementary Figure 12**).

To provide evidence of the phenotypic changes at the genomic level, we analyzed the expression of specific genes previously described as being prominently expressed in M-LECP (44, 45). Quantification of gene expression by qPCR demonstrated that fruquintinib potently upregulated the expression of the anti-inflammatory cytokine *IL-10*, the proinflammatory mediators IL-6 and IL-1β. At the same time, it downregulated the expression of the VEGFR3/FLT4/CD310 ligand, *Vegfc* (**Figure 5D**).

Collectively, these findings demonstrate that fruquintinib dampens myeloid cell polarization towards M-LECP cells, suggesting this cell as another important target in the tumor stroma.

### 3.5 Fruquintinib attenuates nab-PTX–driven polarization of myeloid lymphatic endothelial progenitors from bone marrow macrophages and contributes to improved response to chemotherapy in colon carcinoma

**Figure 6:**
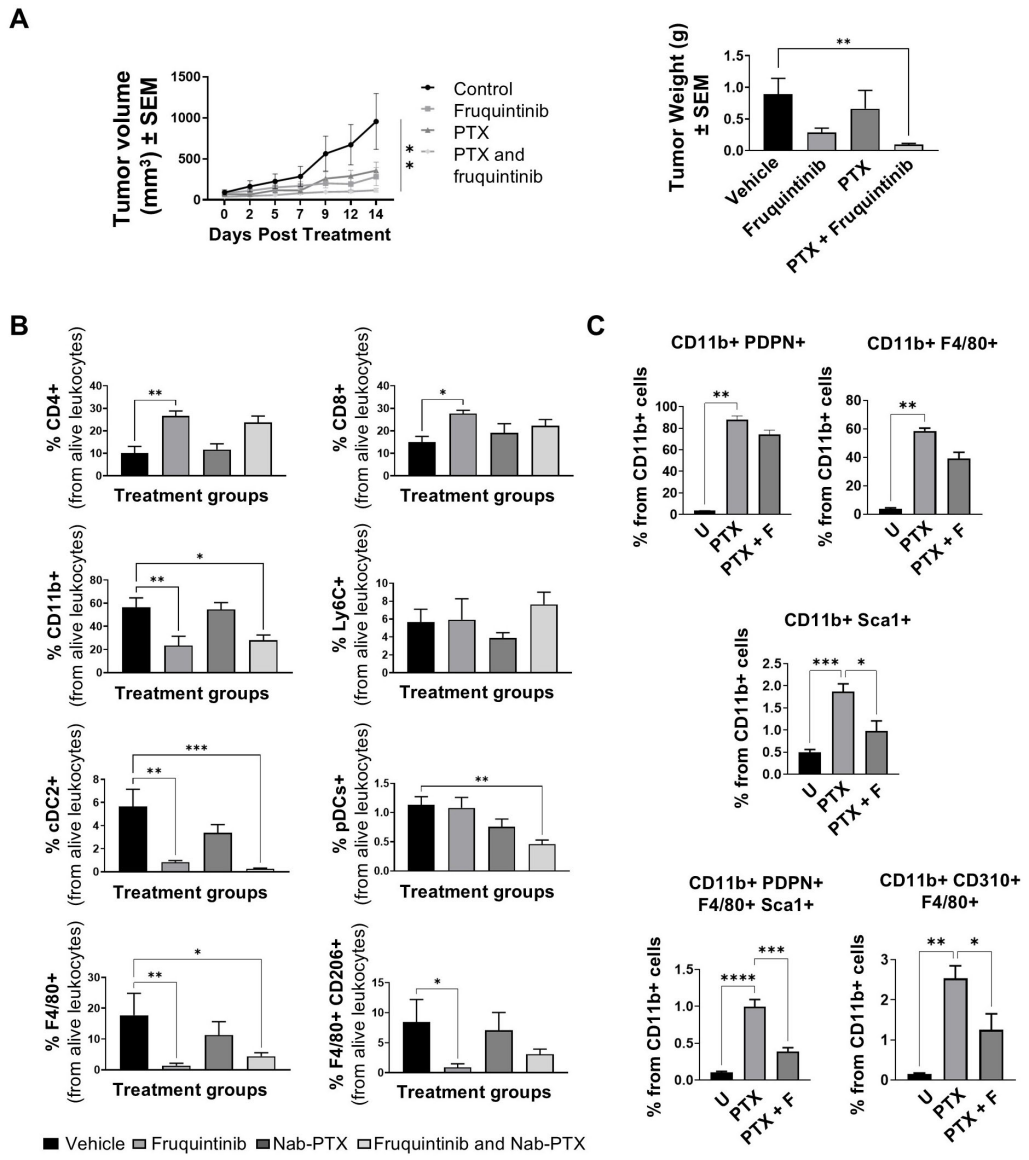
Combinatorial treatment with nab-paclitaxel and fruquintinib enhances the response to single therapy in colon carcinoma models, exhibiting superior myeloid cell depletion compared to nab-paclitaxel alone. **(A-B)** Evolution of tumor volume (left) and tumor weight (right) of MC38 tumor-bearing mice treated for 13 days with fruquintinib (5 mg/kg/day, orally) and/or nab-paclitaxel (10 mg/kg, intravenous) for a total of five doses (days 0, 2, 5, 7, 9 post-treatment). Data corresponding to a single experiment of *n*=6–7 mice per group. **(B)** Quantification by flow cytometry of the frequency of CD4^+^ and CD8^+^ T lymphocytes, CD11b^+^ myeloid cells, Ly6C^+^ inflammatory monocytes, CD4^-^ CD8^-^ CD11b^+^ CD11c^+^ CDc2 cells, CD4^-^ CD8^-^ CD11b^-^ CD11c^+^ plasmacytoid dendritic cells (pDCs), F4/80^+^ macrophages and, immunosuppressive F4/80^+^CD206^+^ macrophages in mice treated with fruquintinib, nab-paclitaxel or a combination of both as in **(A)**. **(C)** Quantification by flow cytometry of podoplanin-positive myeloid cells (CD11b^+^ PDPN^+^), macrophages (CD11b^+^ F4/80^+^), CD11b stem cells (CD11b^+^Sca1^+^), M-LECP (CD11b^+^ PDPN^+^ F4/80^+^ Sca1^+^) and VEGFR3/FLT4/CD310 expressing macrophages (CD11b^+^ CD310^+^, F4/80^+^), in *ex vivo* differentiated M-LECP from bone marrow-derived cells treated with nab-PTX (30 nM) or nab-PTX + fruquintinib (5 µM), (three independent experiments of n=2 mice per condition). (U, untreated; PTX: Nab-paclitaxel; PTX+F: Nab-paclitaxel and fruquintinib). Data are presented as mean ± SEM. Statistical analyses were performed using one-way ANOVA. *p < 0.05 **p < 0.01 ***p < 0.001 and ****p < 0.0001.

It has been shown that the induction of TLR4 activation may promote resistance to nab-paclitaxel (nab-PTX) through the activation of the VEGFR3/FLT4/CD310 pathway (44, 56, 57). With this in mind, we assessed whether combining fruquintinib with nab-PTX could enhance therapeutic outcomes in the murine MC38 colon carcinoma model and whether this could be mediated through inhibition of M-LEPC differentiation. MC38 tumor-bearing mice were treated daily with fruquintinib (5 mg/kg) for 13 days and received five doses of nab-PTX (10 mg/kg) every two or three days. Tumor volume and weight were monitored to assess therapeutic efficacy. As shown in **Figure 6A**, the combined treatment with nab-PTX and fruquintinib resulted in the most significant reduction in tumor growth and weight compared to vehicle or monotherapy (*p <* 0.01). The second-best treatment outcome was observed in the fruquintinib-treated group (*p <*0.05). Immune profiling by flow cytometry (**Figure 6B**) revealed that fruquintinib, as expected, significantly increased the infiltration of CD4^+^ (*p <* 0.01) and CD8^+^ T cells (*p <* 0.05), and reduced the overall population of CD11b^+^ myeloid cells (*p <* 0.01) compared to the control group. In contrast, treatment with nab-PTX alone did not significantly alter either lymphoid or myeloid populations. Importantly, compared to nab-PTX monotherapy, the combination therapy further reduced several myeloid subsets (*p <* 0.01), including cDC2 (CD4^-^ CD8^-^ CD11b^+^CD11c^+^IAb^+^) dendritic cells, plasmacytoid dendritic cells pDCs (CD4^-^ CD8^-^ CD11b^-^ CD11c^+^), and macrophages (F4/80^+^ CD206^+^).

Finally, we studied whether the inhibition of the VEGFR3/FLT4/CD310 receptor with fruquintinib treatment impaired the nab-PTX-mediated differentiation of M-LECP from bone marrow precursors. Bone marrow-derived cells were seeded on fibronectin-coated plates and treated with M-CSF (10 ng/mL) for six days, being stimulated at day 3 with nab-PTX (30 nM) or nab-PTX plus fruquintinib (5 µM). Cells were harvested on day 6. Flow cytometric analysis showed that nab-PTX markedly increased the frequency of CD11b^+^ cells expressing the M-LECP markers PDPN, F4/80, and Sca1. Notably, combined treatment with fruquintinib significantly reduced the nab-PTX-induced upregulation of these surface markers, indicating that fruquintinib attenuates nab-PTX-driven macrophage activation and phenotypic polarization towards M-LECP (**Figure 6C**).

### 3.6 VEGFR3/FLT4 may serve as a prognostic factor for colon carcinoma patients

**Figure 7:**
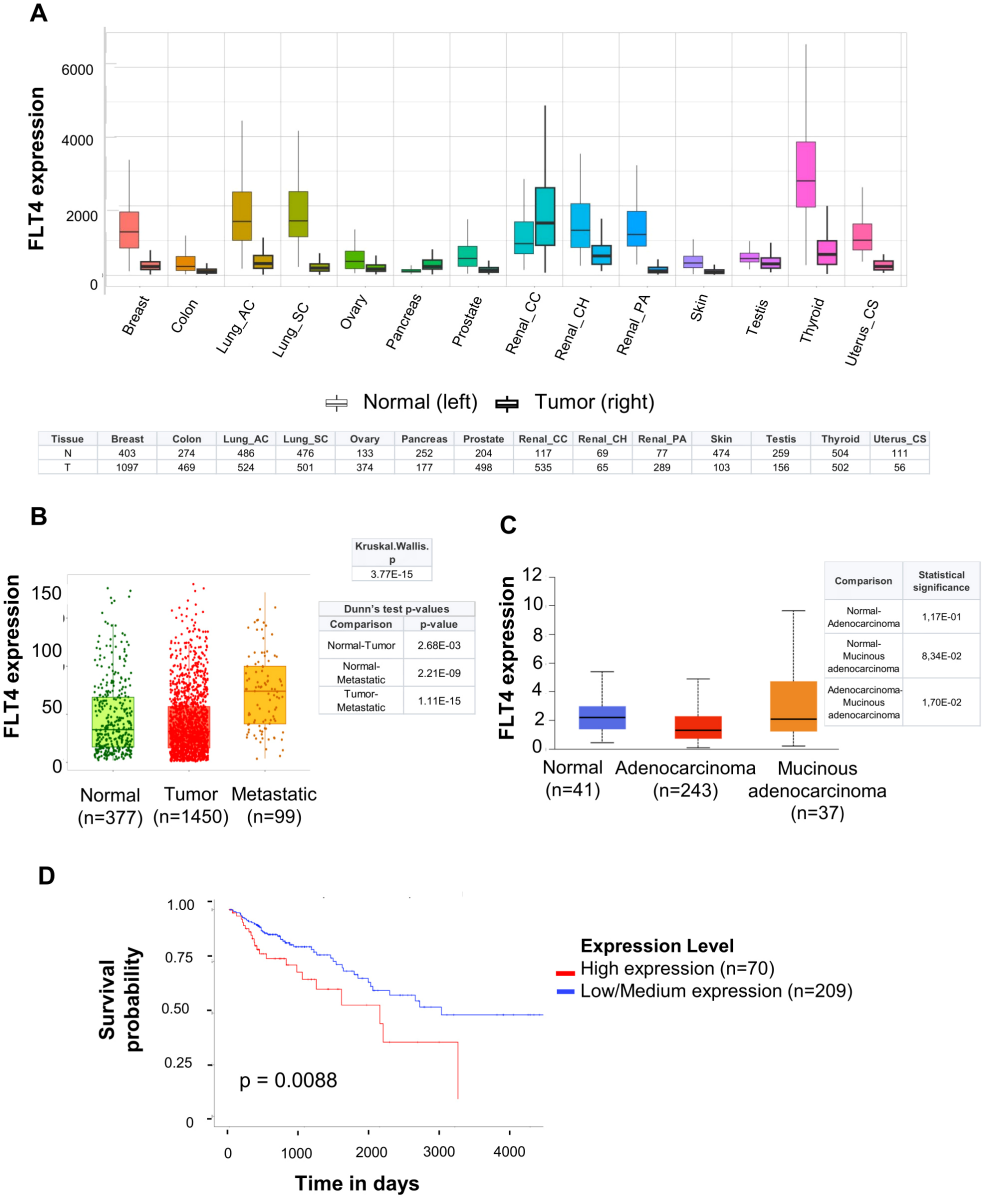
In silico transcriptomic analysis of FLT4 (VEGFR3/CD310) expression using TNMPlot and UALCAN human cancer databases shows an association between FLT4 expression, metastases, and poorer survival of colon cancer patients. **(A)** Comparison of *FLT4* (VEGFR3/CD310) expression levels between normal and primary tumor tissues across multiple cancer types using TNMplot (https://tnmplot.com). Statistical significance was assessed using the Mann-Whitney U test (*p* < 0.05). Sample sizes are presented in the table below the graph. N Normal; T Tumor. **(B)** Gene chip expression of *FLT4* in normal colon tissue, primary colon tumors, and colon cancer metastases samples on the TNMplot platform. Samples were analyzed separately by the Kruskal-Wallis test followed by Dunn’s multiple comparisons test. Sample sizes and statistical analysis are detailed above and beside the graph. **(C)***FLT4* expression across histological subtypes of colon cancer samples analyzed using UALCAN (http://ualcan.path.uab.edu). The boxplots illustrate significant differences in FLT4 expression. Statistical analyses were performed using the methods implemented within the UALCAN platform. Corresponding p-values for group comparisons are presented adjacent to the graph. Sample sizes for each subtype are indicated alongside the figure. **(D)** Kaplan-Meier survival analysis depicting overall survival of colorectal cancer patients stratified by low and high *FLT4* expression levels in a series of colon carcinoma patients from the UALCAN platform. Statistical significance of survival differences was assessed using the log-rank test. The results from each search have been represented as they appeared in the TNMplot and UALCAN platforms, with only minor aesthetic modifications (table format, and font size).

Finally, to support our results with preliminary clinical evidence, we analyzed publicly available datasets using platforms such as TNMplot and UALCAN to investigate whether FLT4 (VEGFR3/CD310) expression could serve as a robust biomarker for stratifying colorectal carcinoma patients suitable for treatment with fruquintinib. Unexpectedly, FLT4 mRNA expression was reduced in a significant number of tumors compared to normal tissues across multiple organs, including the colon and breast carcinoma (**Figure 7A**). In contrast, we observed increased FLT4 expression in metastatic samples (compared to healthy tissue or tumoral samples) and in more aggressive histological mucinous subtype from colon carcinoma patients (**Figures 7B, C**). Moreover, we observed increased survival in samples from colon carcinoma patients with low FLT4 expression (**Figure 7D**).

These preliminary data suggest that VEGFR3/FLT4/CD310 may be a potential prognostic biomarker for colorectal carcinoma, warranting further clinical investigation to validate these findings.

## 4 Discussion

Fruquintinib has emerged as a specific VEGFR3/FLT4/CD310 inhibitor that potently inhibits angiogenesis and tumor growth in preclinical models (32) and clinical trials (58) of colon carcinoma, with recent approvals for its use as a third-line therapy in China, the USA, Canada, and several European countries. In this work, besides corroborating its effects in models of colon carcinoma, we extend its impact to two syngeneic murine breast carcinoma models (4T1 and E0771 cells). Moreover, we demonstrate a reduction in lymph node and pulmonary metastases after treatment with fruquintinib. In addition, we provide *in vitro* and *in vivo* evidence of the efficacy of fruquintinib to target blood and lymphatic vessel formation. These results are consistent with studies using other, less specific VEGFR-3 inhibitors, such as SAR131675 in breast xenograft or syngeneic mouse tumor models (59) and AD0157 (60), which were unable to reach the clinic due to their elevated toxicities. Clinical evidence of the efficacy of fruquintinib in breast cancer is not yet available, as the only open clinical trial in the USA, promoted by a Chinese pharmaceutical company (Hutchinsons Med, NCT04577963) (61), was prematurely closed due to strategic reasons. Nevertheless, targeting VEGFR3/FLT4/CD310 holds promise for inhibiting breast cancer metastasis.

Accumulating evidence indicates that VEGFR3 inhibitors, besides targeting the vascular compartment, may affect other cells of the tumor stroma, particularly immune cells. A seminal study from Espagnolle and collaborators (62) demonstrated that treatment with the VEGFR3 inhibitor SAR131675 reduced MDSCs in lymphoid organs as well as F4/80^High^ populations in tumors. In addition, Li and co-workers (33) demonstrated that the combination of fruquintinib with the anti-PD-1 antibody sintilimab synergistically inhibited colorectal cancer growth by remodeling the immune landscape within tumors, including increased infiltration and activation of cytotoxic T cells, and a reduction in immunosuppressive (CD11b^+^ Gr1^+^) cell populations. Moreover, fruquintinib has been shown to reduce regulatory T cell populations in a B16-OVA tumor model, further contributing to the alleviation of immunosuppression in the tumor microenvironment and enhancing antitumor immune responses (63). Whether these effects are restricted to changes in blood and lymphatic vascular permeability, which may alter the ingress or egress of immune cells, or due to direct targeting of the immune compartment, remains unclear.

In this work, we present new evidence on the direct effect of fruquintinib on myeloid cells in the context of tumor biology. Here, we provided data showing evident VEGFR3/FLT4/CD310 expression in different subpopulations of the myeloid compartment of the tumor stroma and in the bone marrow of tumor-bearing and non-tumor-bearing mice. Our results point to monocytic-myeloid cells as the preferential VEGFR3-expressing compartment, while it is barely detected in granulocytes and only minimally in T lymphocytes.

Hamrah et al. demonstrated that corneal dendritic cells express VEGFR3/FLT4/CD310, particularly during inflammation, identifying them as immature monocytic cells with low levels of MHC-II, CD80, and CD86 (64). In this sense, Fernandez Pujol et al. showed how VEGFR3/FLT4/CD310 expression in immature DCs endowed them with endothelial features under angiogenic stimulation, illustrating their functional plasticity (65). Interestingly, VEGFR3/FLT4/CD310 expression has been associated with the differentiation of a specialized population termed myeloid-lymphatic endothelial cell progenitor (M-LECP) characterized by the co-expression of markers of myeloid progenitors (CD11b, Sca1) and lymphatic endothelial cells (LYVE-1, PDPN) (66). Importantly, these cells are capable of intercalating and fusing with the existing lymphatic vessels, contributing to lymphangiogenesis (67, 68). Therefore, the expression of VEGFR3/FLT4/CD310 on myeloid progenitors may serve as the touchstone that determines the differentiation of myeloid precursors towards mature myeloid populations or endothelial phenotypes.

In our hands, treatment with the VEGFR3/FLT4/CD310 inhibitor fruquintinib as a single agent reshaped the tumor’s immune landscape by incrementing the amount of CD4^+^ and CD8^+^ T lymphocytes, and more importantly, the amounts of immature monocytes (Ly6C^+^) while it reduced the percentages of differentiated myeloid populations, including M2-like (CD206^+^) macrophages and type II dendritic cells (cDC2). These results support the notion that the activation of the VEGFR3/FLT4/CD310 receptor may constitute a limiting step during myeloid cell differentiation. Of note, fruquintinib treatment did not affect granulocyte populations (Ly6G^+^ cells), confirming that this myeloid subpopulation is not a target of VEGFR3/FLT4/CD310 inhibition. In addition, this modulation of the immune milieu in the tumors may help to curb the immune suppression since cDC2 (69) and M2 macrophages (70) play an established immunosuppressive role in tumors.

The effects observed were not limited to the tumoral context but were also observed in the bone marrow of the same mice, suggesting a systemic effect of this drug that affects not only the tumor milieu but also the bone-marrow myeloid compartment. In this sense, we observed a diminution in macrophage-derived endothelial cell progenitors in the bone marrow in mice treated with fruquintinib, along with an accumulation of Ly6C^+^ immature cells, as occurred in tumors. *In vitro* assays confirmed that VEGFR3/FLT4/CD310 inhibition with fruquintinib precluded the differentiation of M-LECP from bone marrow precursors by incrementing instead the production of pro-inflammatory cytokines such as IL-1β and IL-6, which indicates a possible reprogramming towards pro-inflammatory phenotypes with anti-tumoral function (71, 72).

Early studies on tumor angiogenesis demonstrated that bone marrow-derived cells can contribute to tumor angiogenesis and cancer metastases (73, 74). In elegant experiments of bone marrow transplantation and reconstitution, as well as genetic tracing, it was also demonstrated that myeloid precursors integrate into tumor-associated lymphatic vessels (75). Further research into the nature of the pro-lymphangiogenic myeloid cells described them as macrophage-lymphatic endothelial cell progenitors (M-LECP) capable of undergoing lymphatic differentiation upon stimulation with an inflammatory stimulus such as LPS (66), radiation (76), wounding (77), or corneal surgery (78). These cells are capable of incorporating into the endothelial layer of small capillaries at different percentages (67). Notably, the formation of these cells requires transient expression and activation of VEGFR3 and occurs primarily in the bone marrow (42). This cell population may well coincide with the myeloid precursors identified by other groups as BMDCs, which also express lymphatic endothelial cell markers and accumulate at the tips of lymphangiogenic sprouts (75).

In our MC38 tumor model, we observed that diminution of these myeloid precursors in mice treated with fruquintinib was concomitant with impaired tumor vascularization, suggesting that this drug can prevent tumor angiogenesis and lymphangiogenesis not only through direct inhibition of VEGFR3/FLT4/CD310 signaling in endothelial cells, but also by inhibiting macrophage polarization to M-LECPs and their incorporation into endothelial sprouts under angiogenic contexts.

It has been widely described that chemotherapy, in addition to its cytotoxic activity, induces responses that facilitate tumor growth and metastasis, precluding the anti-tumor efficacy of these drugs (79). In this context, taxanes and other chemotherapeutic agents activate the TLR4 signaling pathway in tumor cells, leading to increased VEGFC expression and drug resistance (57). Notably, it has been demonstrated that activation of the VEGFR3/FLT4/CD310 axis in tumor macrophages can promote resistance to taxanes by inducing VEGFC expression (24). Interestingly, this TLR4–VEGFC–VEGFR3 signaling cascade induces the macrophage-derived lymphatic endothelial cell progenitor (M-LECP)-like phenotype (44). To close the circle, M-LECP has been associated with tumor adaptation to chemotherapy (57).

Having this in mind, we evaluated the combined treatment with nab-PTX and fruquintinib to enhance the efficacy of chemotherapy by reducing M-LECP in tumors. *In vivo* assays demonstrated this extreme in a mouse model of colorectal carcinoma, with concomitant increases in lymphocyte infiltration and reductions in myeloid cells similar to those observed with fruquintinib monotherapy. We also demonstrated in ex vivo differentiation assays that nab-PTX promotes polarization of myeloid cells towards pro-lymphatic phenotypes, whereas fruquintinib effectively inhibits this polarization by reducing typical markers of these cells, including F4/80^+^, PDPN^+^, and Sca1^+^ in bone marrow-derived cells.

Although the combination of fruquintinib with immunotherapy has shown promising results in preclinical and clinical trials (80), its combination with chemotherapy is only beginning to be explored. Preclinical studies have demonstrated that fruquintinib combined with trifluridine/tipiracil produces greater antitumor efficacy in colorectal cancer xenografts compared to monotherapy (81). Similarly, enhanced antitumor activity has been observed in PDX models combining fruquintinib with docetaxel in gastric cancer and with oxaliplatin in colon cancer (32). Our results support fruquintinib as a plausible candidate for combination with nab-PTX in cancer treatment. The phase III FRUTIGA trial (NCT03223376) evaluated fruquintinib plus paclitaxel as second-line therapy for advanced gastric cancer, demonstrating a significant improvement in progression-free survival (PFS: 5.6 *vs*. 2.7 months). However, no overall survival benefit was observed (OS: 9.6 *vs*. 8.4 months) (82). To date, there is no evidence for combining nab-PTX with fruquintinib in the treatment of colorectal or breast carcinoma patients.

To provide preliminary evidence on the possible use of VEGFR3/FLT4/CD310 as a prognostic marker in colon carcinoma, we analyzed publicly available gene expression datasets. Although FLT4 was generally downregulated in primary colorectal tumors compared to normal tissue, it caught our attention that its expression is elevated in metastatic lesions and is associated with aggressive histological features such as mucinous tumors. These associations confirm the role of VEGFR3/FLT4/CD310 in tumor progression and dissemination. Moreover, elevated VEGFR3/FLT4/CD310 levels are associated with poorer overall survival in two small exploratory series of colon carcinoma patients. These results are consonant with clinical trials such as the pivotal FRESCO and FRESCO-2 studies, where fruquintinib achieved significant improvements in progression-free and overall survival in patients with refractory metastatic colorectal cancer (35). In sum, these findings provide evidence that FLT4 (VEGFR3/CD310) expression could serve as a valuable prognostic biomarker to stratify colorectal cancer patients and select those who will likely respond to fruquintinib-based treatments. But still, there is an imperative need for additional clinical studies to validate this predictive potential.

Collectively, our data highlight the dual antiangiogenic and immunomodulatory properties of fruquintinib, supporting its use in combinatorial treatment strategies aimed at targeting both the tumor vasculature and tumor immune evasion mechanisms. Future studies are warranted to elucidate the molecular pathways underlying fruquintinib’s immunomodulatory effects and to validate these findings in clinical settings.

## Data Availability

The original contributions presented in the study are included in the article/**Supplementary Material**. Further inquiries can be directed to the corresponding author.

## References

[1] Breslin JW Yang Y Scallan JP Sweat RS Adderley SP Murfee WL . Lymphatic vessel network structure and physiology. Compr Physiol. (2018) 9:207–99. doi: 10.1002/cphy.c180015, PMID: 30549020 PMC6459625

[2] Dieterich LC Detmar M . Tumor lymphangiogenesis and new drug development. Adv Drug Delivery Rev. (2016) 99:148–60. doi: 10.1016/j.addr.2015.12.011, PMID: 26705849

[3] Malekan M Haass NK Rokni GR Gholizadeh N Ebrahimzadeh MA Kazeminejad A . VEGF/VEGFR axis and its signaling in melanoma: Current knowledge toward therapeutic targeting agents and future perspectives. Life Sci. (2024) 345:122563. doi: 10.1016/j.lfs.2024.122563, PMID: 38508233

[4] Brogowska KK Zajkowska M Mroczko B . Vascular endothelial growth factor ligands and receptors in breast cancer. J Clin Med. (2023) 12:2412. doi: 10.3390/jcm12062412, PMID: 36983412 PMC10056253

[5] Huang C Chen Y . Lymphangiogenesis and colorectal cancer. Saudi Med J. (2017) 38:237–44. doi: 10.15537/smj.2017.3.16245, PMID: 28251217 PMC5387898

[6] Liu P Ding P Sun C Chen S Lowe S Meng L . Lymphangiogenesis in gastric cancer: function and mechanism. Eur J Med Res. (2023) 28:405. doi: 10.1186/s40001-023-01298-x, PMID: 37803421 PMC10559534

[7] Ran S Volk-Draper L . Lymphatic endothelial cell progenitors in the tumor microenvironment. Adv Exp Med Biol. (2020) 1234:87–105. doi: 10.1007/978-3-030-37184-5_7, PMID: 32040857 PMC7263265

[8] Wei R Lv M Li F Cheng T Zhang Z Jiang G . Human CAFs promote lymphangiogenesis in ovarian cancer via the Hh-VEGF-C signaling axis. Oncotarget. (2017) 8:67315–28. doi: 10.18632/oncotarget.18621, PMID: 28978035 PMC5620175

[9] Maisel K Sasso MS Potin L Swartz MA . Exploiting lymphatic vessels for immunomodulation: Rationale, opportunities, and challenges. Adv Drug Delivery Rev. (2017) 114:43–59. doi: 10.1016/j.addr.2017.07.005, PMID: 28694027 PMC6026542

[10] Hirosue S Vokali E Raghavan VR Rincon-Restrepo M Lund AW Corthésy-Henrioud P . Steady-state antigen scavenging, cross-presentation, and CD8^+^ T cell priming: a new role for lymphatic endothelial cells. J Immunol. (2014) 192:5002–11. doi: 10.4049/jimmunol.1302492, PMID: 24795456 PMC4025611

[11] Lukacs-Kornek V Malhotra D Fletcher AL Acton SE Elpek KG Tayalia P . Regulated release of nitric oxide by nonhematopoietic stroma controls expansion of the activated T cell pool in lymph nodes. Nat Immunol. (2011) 12:1096–104. doi: 10.1038/ni.2112, PMID: 21926986 PMC3457791

[12] Nörder M Gutierrez MG Zicari S Cervi E Caruso A Guzmán CA . Lymph node-derived lymphatic endothelial cells express functional costimulatory molecules and impair dendritic cell-induced allogenic T-cell proliferation. FASEB J. (2012) 26:2835–46. doi: 10.1096/fj.12-205278, PMID: 22459150

[13] Podgrabinska S Kamalu O Mayer L Shimaoka M Snoeck H Randolph GJ . Inflamed lymphatic endothelium suppresses dendritic cell maturation and function via Mac-1/ICAM-1-dependent mechanism. J Immunol. (2009) 183:1767–79. doi: 10.4049/jimmunol.0802167, PMID: 19587009 PMC4410990

[14] Kannan S Rutkowski JM . VEGFR-3 signaling in macrophages: friend or foe in disease? Front Immunol. (2024) 15:1349500. doi: 10.3389/fimmu.2024.1349500, PMID: 38464522 PMC10921555

[15] Kuonqui KG Campbell AC Pollack BL Shin J Sarker A Brown S . Regulation of VEGFR3 signaling in lymphatic endothelial cells. Front Cell Dev Biol. (2025) 13:1527971. doi: 10.3389/fcell.2025.1527971, PMID: 40046235 PMC11880633

[16] Kuonqui K Campbell AC Sarker A Roberts A Pollack BL Park HJ . Dysregulation of lymphatic endothelial VEGFR3 signaling in disease. Cells. (2023) 13:68. doi: 10.3390/cells13010068, PMID: 38201272 PMC10778007

[17] Shigeta K Hato T Chen Y Duda DG . Anti-VEGFR therapy as a partner for immune-based therapy approaches in HCC. In: Greten TF , editor. Immunotherapy of hepatocellular carcinoma. Springer International Publishing, Cham (2017). p. 85–101.

[18] Keating GM . Axitinib: a review in advanced renal cell carcinoma. Drugs. (2015) 75:1903–13. doi: 10.1007/s40265-015-0483-x, PMID: 26487541

[19] Li H Huang H Zhang T Feng H Wang S Zhang Y . Apatinib: A novel antiangiogenic drug in monotherapy or combination immunotherapy for digestive system Malignancies. Front Immunol. (2022) 13:937307. doi: 10.3389/fimmu.2022.937307, PMID: 35844616 PMC9276937

[20] Dai S Zhong Y Cui H Zhao J Li S . Aortic dissection induced by vascular endothelial growth factor inhibitors. Front Pharmacol. (2023) 14:1189910. doi: 10.3389/fphar.2023.1189910, PMID: 37426822 PMC10327890

[21] Yao SX Huang YJ Zhang YX Cui ZX Lu HY Wang R . Revisiting VEGF/VEGFR-2 signalling as an anticancer target and its inhibitor discovery: where are we and where should we go? J Drug Target. (2025) 33(9):1471–94. doi: 10.1080/1061186X.2025.2508985, PMID: 40387416

[22] Shibata MA Shibata E Tanaka Y Shiraoka C Kondo Y . Soluble Vegfr3 gene therapy suppresses multi-organ metastasis in a mouse mammary cancer model. Cancer Sci. (2020) 111:2837–49. doi: 10.1111/cas.14531, PMID: 32539229 PMC7419054

[23] Hsu MC Pan MR Hung WC . Two birds, one stone: double hits on tumor growth and lymphangiogenesis by targeting vascular endothelial growth factor receptor 3. Cells. (2019) 8:270. doi: 10.3390/cells8030270, PMID: 30901976 PMC6468620

[24] Alishekevitz D Gingis-Velitski S Kaidar-Person O Gutter-Kapon L Scherer SD Raviv Z . Macrophage-induced lymphangiogenesis and metastasis following paclitaxel chemotherapy is regulated by VEGFR3. Cell Rep. (2016) 17:1344–56. doi: 10.1016/j.celrep.2016.09.083, PMID: 27783948 PMC5098117

[25] Dumond A Montemagno C Vial V Grépin R Pagès G . Anti-vascular endothelial growth factor C antibodies efficiently inhibit the growth of experimental clear cell renal cell carcinomas. Cells. (2021) 10:1222. doi: 10.3390/cells10051222, PMID: 34067671 PMC8157203

[26] Saif MW Knost JA Chiorean EG Kambhampati SR Yu D Pytowski B . Phase 1 study of the anti-vascular endothelial growth factor receptor 3 monoclonal antibody LY3022856/IMC-3C5 in patients with advanced and refractory solid tumors and advanced colorectal cancer. Cancer Chemother Pharmacol. (2016) 78:815–24. doi: 10.1007/s00280-016-3134-3, PMID: 27566701

[27] Galvagni F Pennacchini S Salameh A Rocchigiani M Neri F Orlandini M . Endothelial cell adhesion to the extracellular matrix induces c-Src-dependent VEGFR-3 phosphorylation without the activation of the receptor intrinsic kinase activity. Circ Res. (2010) 106:1839–48. doi: 10.1161/CIRCRESAHA.109.206326, PMID: 20431062

[28] Alam A Blanc I Gueguen-Dorbes G Duclos O Bonnin J Barron P . SAR131675, a potent and selective VEGFR-3-TK inhibitor with antilymphangiogenic, antitumoral, and antimetastatic activities. Mol Cancer Ther. (2012) 11:1637–49. doi: 10.1158/1535-7163.MCT-11-0866-T, PMID: 22584122

[29] Walsh KA Kastrappis G Fifis T Paolini R Christophi C Perini MV . SAR131675, a VEGRF3 inhibitor, modulates the immune response and reduces the growth of colorectal cancer liver metastasis. Cancers (Basel). (2022) 14:2715. doi: 10.3390/cancers14112715, PMID: 35681695 PMC9179346

[30] Paillasse MR Esquerré M Bertrand FA Poussereau-Pomié C Pichery M Visentin V . Targeting tumor angiogenesis with the selective VEGFR-3 inhibitor EVT801 in combination with cancer immunotherapy. Cancer Res Commun. (2022) 2:1504–19. doi: 10.1158/2767-9764.CRC-22-0151, PMID: 36970050 PMC10035370

[31] Patell K Mears VL Storandt MH Mahipal A . Metabolism, toxicity and management of fruquintinib: a novel drug for metastatic colorectal cancer. Expert Opin Drug Metab Toxicol. (2024) 20:197–205. doi: 10.1080/17425255.2024.2332364, PMID: 38497279

[32] Sun Q Zhou J Zhang Z Guo M Liang J Zhou F . Discovery of fruquintinib, a potent and highly selective small molecule inhibitor of VEGFR 1, 2, 3 tyrosine kinases for cancer therapy. Cancer Biol Ther. (2014) 15:1635–45. doi: 10.4161/15384047.2014.964087, PMID: 25482937 PMC4622458

[33] Li Q Cheng X Zhou C Tang Y Li F Zhang B . Fruquintinib enhances the antitumor immune responses of anti-programmed death receptor-1 in colorectal cancer. Front Oncol. (2022) 12:841977. doi: 10.3389/fonc.2022.841977, PMID: 35371995 PMC8968679

[34] Tan S Zhang S Zhou N Cai X Yi C Gou H . Efficacy and safety of fruquintinib dose-escalation strategy for elderly patients with refractory metastatic colorectal cancer: A single-arm, multicenter, phase II study. Cancer Med. (2023) 12:22038–46. doi: 10.1002/cam4.6786, PMID: 38063405 PMC10757135

[35] Dasari A Lonardi S Garcia-Carbonero R Elez E Yoshino T Sobrero A . Fruquintinib versus placebo in patients with refractory metastatic colorectal cancer (FRESCO-2): an international, multicentre, randomised, double-blind, phase 3 study. Lancet. (2023) 402:41–53. doi: 10.1016/S0140-6736(23)00772-9, PMID: 37331369

[36] Shirley M . Fruquintinib: first global approval. Drugs. (2018) 78:1757–61. doi: 10.1007/s40265-018-0998-z, PMID: 30357594

[37] Fusco MJ Casak SJ Mushti SL Cheng J Christmas BJ Thompson MD . FDA approval summary: fruquintinib for the treatment of refractory metastatic colorectal cancer. Clin Cancer Res. (2024) 30:3100–4. doi: 10.1158/1078-0432.CCR-24-0281, PMID: 38809262 PMC11293994

[38] Canadian Journal of Health Technologies . Fruquintinib (Fruzaqla) (2024). Available online at: https://canjhealthtechnol.ca/index.php/cjht/article/view/PC0352/2286 (Accessed September 1, 2025).

[39] Therapeutic Goods Administration . FRUZAQLA fruquintinib 5 mg hard capsule bottle. Public Summary for ARTG Entry 422038 (2024). ARTG ID422038. Available online at: https://www.ebs.tga.gov.au/servlet/xmlmillr6?dbid=ebs/PublicHTML/pdfStore.nsf&docid=422038&agid=%28PrintDetailsPublic%29&actionid=1 (Accessed December 2, 2025).

[40] Lu S Zhou JY Niu XM Zhou JY Jian H Yin HY . Fruquintinib with gefitinib as first-line therapy in patients carrying EGFR mutations with advanced non-small cell lung cancer: a single-arm, phase II study. Transl Lung Cancer Res. (2021) 10:839–54. doi: 10.21037/tlcr-20-1028, PMID: 33718026 PMC7947379

[41] Lu S Chen G Sun Y Sun S Chang J Yao Y . A Phase III, randomized, double-blind, placebo-controlled, multicenter study of fruquintinib in Chinese patients with advanced nonsquamous non-small-cell lung cancer - The FALUCA study. Lung Cancer. (2020) 146:252–62. doi: 10.1016/j.lungcan.2020.06.016, PMID: 32592986

[42] Li J Qin S Xu RH Shen L Xu J Bai Y . Effect of fruquintinib vs placebo on overall survival in patients with previously treated metastatic colorectal cancer: the FRESCO randomized clinical trial. JAMA. (2018) 319:2486–96. doi: 10.1001/jama.2018.7855, PMID: 29946728 PMC6583690

[43] Jones DT Cruz G Lugue MT Heer MS Sai M Pace C . Fruquintinib-induced cerebellar hemorrhage in left-sided descending metastatic colorectal adenocarcinoma: A case report and risk assessment. Cureus. (2024) 16:e68203. doi: 10.7759/cureus.68203, PMID: 39221315 PMC11364494

[44] Volk-Draper LD Hall KL Wilber AC Ran S . Lymphatic endothelial progenitors originate from plastic myeloid cells activated by toll-like receptor-4. PloS One. (2017) 12:e0179257. doi: 10.1371/journal.pone.0179257, PMID: 28598999 PMC5466303

[45] Volk-Draper L Athaiya S Espinosa Gonzalez M Bhattarai N Wilber A Ran S . Tumor microenvironment restricts IL-10 induced multipotent progenitors to myeloid-lymphatic phenotype. PloS One. (2024) 19:e0298465. doi: 10.1371/journal.pone.0298465, PMID: 38640116 PMC11029653

[46] Cossarizza A Chang HD Radbruch A Acs A Adam D Adam-Klages S . Guidelines for the use of flow cytometry and cell sorting in immunological studies (second edition). Eur J Immunol. (2019) 49:1457–973. doi: 10.1002/eji.201970107, PMID: 31633216 PMC7350392

[47] Ruiz-Fernández de Córdoba B Moreno H Valencia K Perurena N Ruedas P Walle T . Tumor ENPP1 (CD203a)/haptoglobin axis exploits myeloid-derived suppressor cells to promote post-radiotherapy local recurrence in breast cancer. Cancer Discov. (2022) 12:1356–77. doi: 10.1158/2159-8290.CD-21-0932, PMID: 35191482 PMC7612709

[48] Stankovic B Bjørhovde HAK Skarshaug R Aamodt H Frafjord A Müller E . Immune cell composition in human non-small cell lung cancer. Front Immunol. (2019) 9:3101. doi: 10.3389/fimmu.2018.03101, PMID: 30774636 PMC6367276

[49] Taylor MA Hughes AM Walton J Coenen-Stass AML Magiera L Mooney L . Longitudinal immune characterization of syngeneic tumor models to enable model selection for immune oncology drug discovery. J Immunother Cancer. (2019) 7:328. doi: 10.1186/s40425-019-0794-7, PMID: 31779705 PMC6883640

[50] Bartha A Győrffy B . TNMplot: differential gene gene expression analysis in Tumor, Normal and Metastatic tissues (2021). Available online at: https://tnmplot.com/analysis/ (Accessed September 2, 2025).

[51] Bartha Á Győrffy B . TNMplot.com: A web tool for the comparison of gene expression in normal, tumor and metastatic tissues. Int J Mol Sci. (2021) 22:2622. doi: 10.3390/ijms22052622, PMID: 33807717 PMC7961455

[52] The University of Alabama . The University of ALabama at Birmingham CANcer data analysis Portal (2017). Available online at: https://ualcan.path.uab.edu/ (Accessed September 1, 2025).

[53] Chandrashekar DS Bashel B Balasubramanya SAH Creighton CJ Ponce-Rodriguez I Chakravarthi BVSK . UALCAN: A portal for facilitating tumor subgroup gene expression and survival analyses. Neoplasia. (2017) 19:649–58. doi: 10.1016/j.neo.2017.05.002, PMID: 28732212 PMC5516091

[54] Chandrashekar DS Karthikeyan SK Korla PK Patel H Shovon AR Athar M . UALCAN: An update to the integrated cancer data analysis platform. Neoplasia. (2022) 25:18–27. doi: 10.1016/j.neo.2022.01.001, PMID: 35078134 PMC8788199

[55] Kukk E Lymboussaki A Taira S Kaipainen A Jeltsch M Joukov V . VEGF-C receptor binding and pattern of expression with VEGFR-3 suggests a role in lymphatic vascular development. Development. (1996) 122:3829–37. doi: 10.1242/dev.122.12.3829, PMID: 9012504

[56] Ran S . The role of TLR4 in chemotherapy-driven metastasis. Cancer Res. (2015) 75:2405–10. doi: 10.1158/0008-5472.CAN-14-3525, PMID: 25998620 PMC4470764

[57] Volk-Draper L Hall K Griggs C Rajput S Kohio P DeNardo D . Paclitaxel therapy promotes breast cancer metastasis in a TLR4-dependent manner. Cancer Res. (2014) 74:5421–34. doi: 10.1158/0008-5472.CAN-14-0067, PMID: 25274031 PMC4185415

[58] Yonatan ER Ruby R Prasetya A Arifin ES . Evaluation of fruquintinib’s efficacy and safety in refractory metastatic colorectal cancer: a systematic review and meta-analysis of phase II and III randomized controlled trials. Clin J Gastroenterol. (2025) 18:11–22. doi: 10.1007/s12328-024-02087-7, PMID: 39704756

[59] Li Y Yang G Zhang J Tang P Yang C Wang G . Discovery, synthesis, and evaluation of highly selective vascular endothelial growth factor receptor 3 (VEGFR3) inhibitor for the potential treatment of metastatic triple-negative breast cancer. J Med Chem. (2021) 64:12022–48. doi: 10.1021/acs.jmedchem.1c00678, PMID: 34351741

[60] García-Caballero M Paupert J Blacher S Van de Velde M Quesada AR Medina MA . Targeting VEGFR-3/-2 signaling pathways with AD0157: a potential strategy against tumor-associated lymphangiogenesis and lymphatic metastases. J Hematol Oncol. (2017) 10:122. doi: 10.1186/s13045-017-0484-1, PMID: 28629427 PMC5477162

[61] ClinicalTrials.gov . A study of fruquintinib in Combination With Tislelizumab in Advanced Solid Tumors (2024). Available online at: https://clinicaltrials.gov/study/NCT04577963 (Accessed September 1, 2025).

[62] Espagnolle N Barron P Mandron M Blanc I Bonnin J Agnel M . Specific Inhibition of the VEGFR-3 tyrosine kinase by SAR131675 reduces peripheral and tumor associated immunosuppressive myeloid cells. Cancers (Basel). (2014) 6:472–90. doi: 10.3390/cancers6010472, PMID: 24589997 PMC3980599

[63] Wang Z Shi X Zhao Y Zhou J Zhang S Wang J . DC101, an anti-VEGFR2 agent, promotes high-endothelial venule formation and immune infiltration versus SAR131675 and fruquintinib. Biochem Biophys Res Commun. (2023) 661:10–20. doi: 10.1016/j.bbrc.2023.04.018, PMID: 37084488

[64] Hamrah P Chen L Zhang Q Dana MR . Novel expression of vascular endothelial growth factor receptor (VEGFR)-3 and VEGF-C on corneal dendritic cells. Am J Pathol. (2003) 163:57–68. doi: 10.1016/S0002-9440(10)63630-9, PMID: 12819011 PMC1868166

[65] Fernandez Pujol B Lucibello FC Zuzarte M Lütjens P Müller R Havemann K . Dendritic cells derived from peripheral monocytes express endothelial markers and in the presence of angiogenic growth factors differentiate into endothelial-like cells. Eur J Cell Biol. (2001) 80:99–110. doi: 10.1078/0171-9335-00136, PMID: 11211940

[66] Hall KL Volk-Draper LD Flister MJ Ran S . New model of macrophage acquisition of the lymphatic endothelial phenotype. PloS One. (2012) 7:e31794. doi: 10.1371/journal.pone.0031794, PMID: 22396739 PMC3292559

[67] Ran S Montgomery KE . Macrophage-mediated lymphangiogenesis: the emerging role of macrophages as lymphatic endothelial progenitors. Cancers (Basel). (2012) 4:618–57. doi: 10.3390/cancers4030618, PMID: 22946011 PMC3430523

[68] Athaiya S Volk-Draper L Cox E Robinson K Zinkevich N Ran S . Bone marrow myeloid-lymphatic progenitors expand tumor lymphatic vasculature through cell fusion. Cancers (Basel). (2025) 17:1804. doi: 10.3390/cancers17111804, PMID: 40507286 PMC12153582

[69] Mazzoccoli L Liu B . Dendritic cells in shaping anti-tumor T cell response. Cancers (Basel). (2024) 16:2211. doi: 10.3390/cancers16122211, PMID: 38927916 PMC11201542

[70] Debacker JM Gondry O Lahoutte T Keyaerts M Huvenne W . The prognostic value of CD206 in solid Malignancies: A systematic review and meta-analysis. Cancers (Basel). (2021) 13:3422. doi: 10.3390/cancers13143422, PMID: 34298638 PMC8305473

[71] Beyranvand Nejad E Labrie C van Elsas MJ Kleinovink JW Mittrücker HW Franken KLMC . IL-6 signaling in macrophages is required for immunotherapy-driven regression of tumors. J Immunother Cancer. (2021) 9:e002460. doi: 10.1136/jitc-2021-002460, PMID: 33879600 PMC8061866

[72] Schaefer E Wu W Mark C Yang A DiGiacomo E Carlton-Smith C . Intermittent hypoxia is a proinflammatory stimulus resulting in IL-6 expression and M1 macrophage polarization. Hepatol Commun. (2017) 1:326–37. doi: 10.1002/hep4.1045, PMID: 29404462 PMC5721395

[73] De Palma M Venneri MA Galli R Sergi Sergi L Politi LS Sampaolesi M . Tie2 identifies a hematopoietic lineage of proangiogenic monocytes required for tumor vessel formation and a mesenchymal population of pericyte progenitors. Cancer Cell. (2005) 8:211–26. doi: 10.1016/j.ccr.2005.08.002, PMID: 16169466

[74] Bron S Henry L Faes-Van’t Hull E Turrini R Vanhecke D Guex N . TIE-2-expressing monocytes are lymphangiogenic and associate specifically with lymphatics of human breast cancer. Oncoimmunology. (2015) 5:e1073882. doi: 10.1080/2162402X.2015.1073882, PMID: 27057438 PMC4801424

[75] Zumsteg A Baeriswyl V Imaizumi N Schwendener R Rüegg C Christofori G . Myeloid cells contribute to tumor lymphangiogenesis. PloS One. (2009) 4:e7067. doi: 10.1371/journal.pone.0007067, PMID: 19759906 PMC2738969

[76] Jiang S Bailey AS Goldman DC Swain JR Wong MH Streeter PR . Hematopoietic stem cells contribute to the lymphatic endothelium. PloS One. (2008) 3:e3812. doi: 10.1371/journal.pone.0003812, PMID: 19043576 PMC2583952

[77] Maruyama K Asai J Ii M Thorne T Losordo DW D’Amore PA . Decreased macrophage number and activation lead to reduced lymphatic vessel formation and contribute to impaired diabetic wound healing. Am J Pathol. (2007) 170:1178–91. doi: 10.2353/ajpath.2007.060018, PMID: 17392158 PMC1829452

[78] Religa P Cao R Bjorndahl M Zhou Z Zhu Z Cao Y . Presence of bone marrow-derived circulating progenitor endothelial cells in the newly formed lymphatic vessels. Blood. (2005) 106:4184–90. doi: 10.1182/blood-2005-01-0226, PMID: 16141354

[79] Shaked Y . Balancing efficacy of and host immune responses to cancer therapy: the yin and yang effects. Nat Rev Clin Oncol. (2016) 13:611–26. doi: 10.1038/nrclinonc.2016.57, PMID: 27118493

[80] Assi T Hachem MA Amine-Hneineh R Moussa T . Combination of immunotherapy and fruquintinib in metastatic colorectal cancer: the key to overcome resistance? Immunotherapy. (2024) 16:1171–3. doi: 10.1080/1750743X.2024.2430173, PMID: 39548753 PMC11760286

[81] Nukatsuka M Fujioka A Nagase H Tanaka G Hayashi H . Evaluation of a novel combination therapy, based on trifluridine/tipiracil and fruquintinib, against colorectal cancer. Chemotherapy. (2023) 68:102–10. doi: 10.1159/000528867, PMID: 36623495 PMC10233702

[82] Wang F Shen L Guo W Liu T Li J Qin S . Fruquintinib plus paclitaxel versus placebo plus paclitaxel for gastric or gastroesophageal junction adenocarcinoma: the randomized phase 3 FRUTIGA trial. Nat Med. (2024) 30:2189–98. doi: 10.1038/s41591-024-02989-6, PMID: 38824242

