# Supplementary material for: Fruquintinib saddles tumor immune tolerance by curbing pro-tumoral immature myeloid cell populations
Source: Front Immunol. 2025 Dec 11;16:1699980. doi: 10.3389/fimmu.2025.1699980 (PMC12738944; doi:10.3389/fimmu.2025.1699980)
Supplement: Supplementary file 1 [file DataSheet1.pdf]

Supplementary Table 1. PCR primer sequences.

Gene	Product Size (bp)	Forward Primer Sequence (5' → 3')	Reverse Primer Sequence (5' → 3')
β actin	194	CGCGTCCACCCGCGAG	CCTGGTGCCTAGGGCG
Vegfc	166	CCATGCACTTGCTGTGCTTC	TCTTTGCCTTCAAAAGCCTTGAC
Il6	251	CAACGATGATGCACTTGCAGA	TGTGACTCCAGCTTATCTCTTGG
Il1b	219	GCCACCTTTTGACAGTGATGAG	AAGGTCCACGGGAAAGACAC
Il10	170	CAGAGAAGCATGGCCCAGAA	GACACCTTGGTCTTGGAGCTTA

Supplementary Table 2. Information on antibodies used for flow cytometry.

Marker / Reagent	Conjugate	Host Species	Clone	Company	Catalog Number
CD11b	PE-Cy7	Rat	M1/70	BioLegend	101215
CD11c	BV510	Armenian Hamster	N418	BioLegend	117337
CD206	BV650	Rat	C068C2	BioLegend	141723
CD310	APC	Rat	REA860	Miltenyi Biotec	130-112-773
CD3	BV605	Rat	17A2	BioLegend	100237
CD3a	APC	Rat	17A2	BioLegend	100311
CD4	BV650	Rat	GK1.5	BioLegend	100545
CD45.2	PerCP-Cy5.5	Rat	104	BioLegend	109827
CD86	AF488	Rat	GL1	BioLegend	105018
CD8a	PE	Rat	53-6.7	BioLegend	100707
CD8a	FITC	Rat	53-6.7	BioLegend	100705
CD8a	BV510	Rat	53-6.7	BioLegend	100751
F4/80	BV421	Rat	BM8	BioLegend	123132
F4/80	PE	Rat	BM8	Invitrogen	123110
I-A/I-E	APC	Rat	M5/114.15.2	BioLegend	107613
Ly6C	AF700	Rat	HK1.4	BioLegend	128024
Ly6G	BV605	Rat	1A8	BioLegend	127639
Ly6G	PE	Rat	1A8	BioLegend	127608
PDPN	BV421	Armenian Hamster	8.1.1	BioLegend	127423
Sca-1 (Ly-6A/E)	BV605	Rat	D7	BioLegend	108133
Zombie NIR	Near-Infrared	—	—	BioLegend	423105

Supplementary Table 3. Information on antibodies used for immunohistochemistry.

Target	Antibody Reference	Manufacturer	Host Species	Dilution	Antigen Retrieval pH	Antibody Type
CD31	HS-351117	HistoSure	Rat	1/500	6	Primary
Ly6C	ab314120	Abcam	Rabbit	1/100	9	Primary
EpCAM	ab213501	Abcam	Rabbit	1/1000	6	Primary
LYVE-1	ab141917	Abcam	Rabbit	1/100	6	Primary
Anti-Rat IgG H&L (HRP)	ab97057	Abcam	Goat	1/100	–	Secondary
Anti-Rabbit Poly-HRP-IgG	K4065	Agilent Technologies	Goat	1/100	–	Secondary

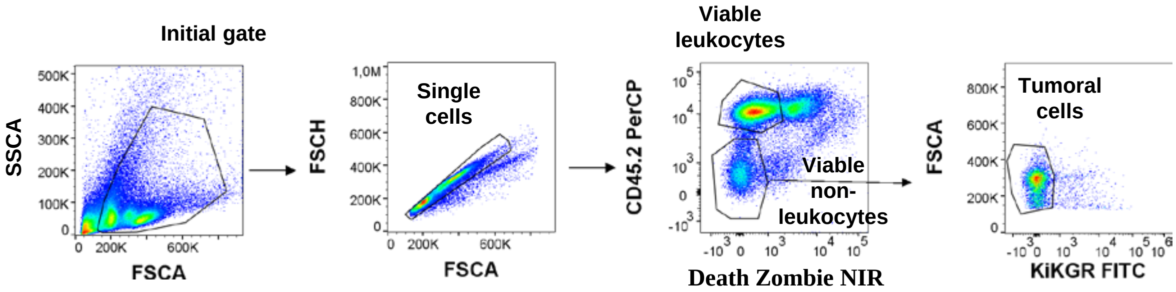

Supplementary Figure 1. Representative flow cytometry gating strategy for detection of metastatic tumoral cells on Kikume mice ($KiKGR^-$) in lymph nodes on Figure 1D.

Cells were initially gated by side and forward scatter (SSCA/FSCA), followed by exclusion of doublets (FSCH/FSCA). Live non-leukocyte cells were identified as $CD45.2^-$ Zombie NIR^-, and tumoral cells were further gated as KiKGR $FITC^-$ events.

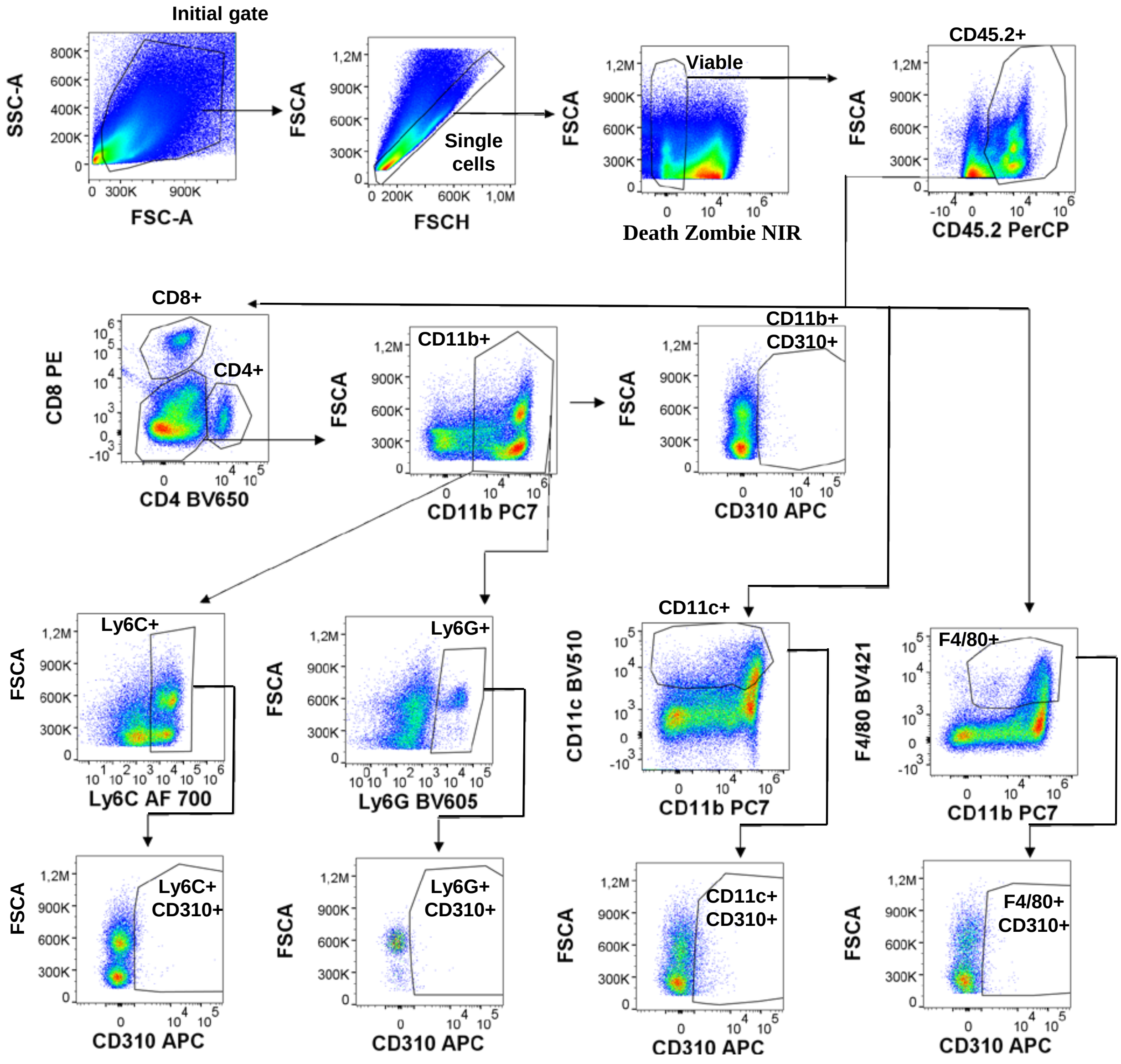

Supplementary Figure 2. Representative flow cytometry gating strategy corresponding to Figure 3 and Figure 4B and C.

Representative flow cytometry gating strategy on tumoral cells from a control mouse. Cells were initially gated based on forward and side scatter (FSC-A vs SSC-A) to define cell size and granularity, followed by exclusion of doublets (FSC-H vs FSC-A) and dead cells (Zombie NIR^{+}). Single live CD45.2^{+} immune cells were analyzed for CD4 and CD8 expression to identify T cell subsets. Myeloid and dendritic cell populations were further characterized, including dendritic cells (CD11b$^{+/-}$ CD11c^{+}), macrophages (CD4^{-} CD8^{-} CD11b^{+} F4/80^{+}), Ly6C^{+} monocytes (CD4^{-} CD8^{-} CD11b^{+} Ly6C^{+}), and neutrophils (CD4^{-} CD8^{-} CD11b^{+} Ly6G^{+}). CD310 expression was assessed across each immune subset in Figure 3.

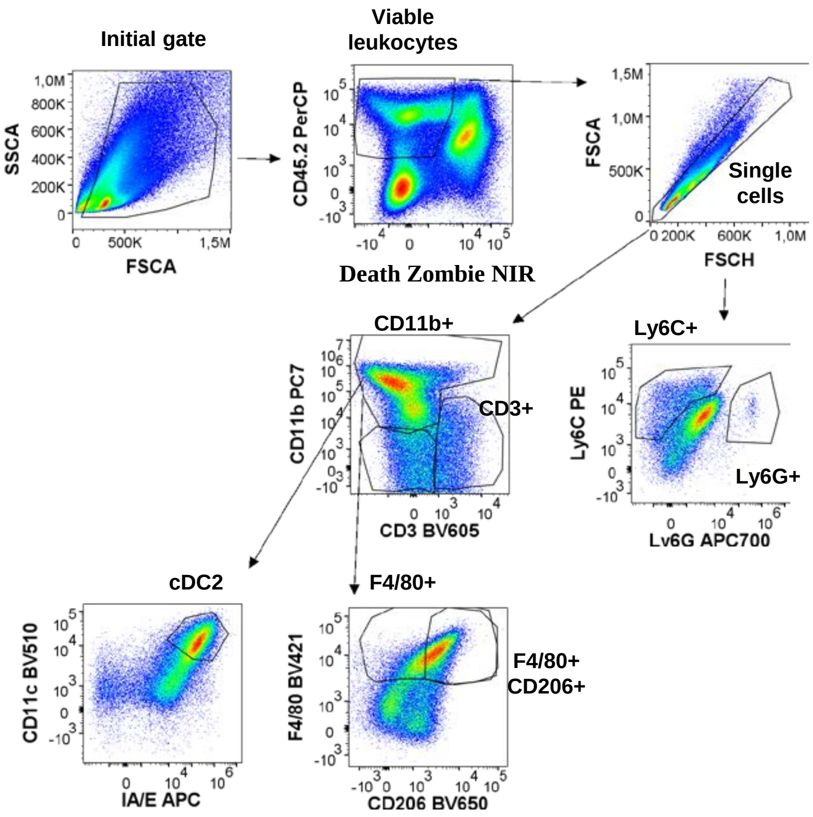

Supplementary Figure 3. Representative flow cytometry gating strategy corresponding to Figure 4D and 4E.

Representative flow cytometry gating strategy on tumoral cells from a control mouse. Initial gating was based on side scatter (SSCA) and forward scatter (FSCA) to define cell size and granularity. Viable leukocytes were identified as $CD45.2^{+}$ Zombie NIR^{-} cells, and single cells were gated by forward scatter height (FSCH) vs. forward scatter area (FSCA). $CD11b^{+}$ subsets were distinguished based on Ly6C and Ly6G expression to discriminate monocytes from neutrophils. Conventional type 2 dendritic cells (cDC2) were identified as $CD11b^{+}$ $CD11c^{+}$ I-A/$I\text{-}E^{+}$, while M2 lyke macrophages were defined by co-expression of F4/80 and CD206.

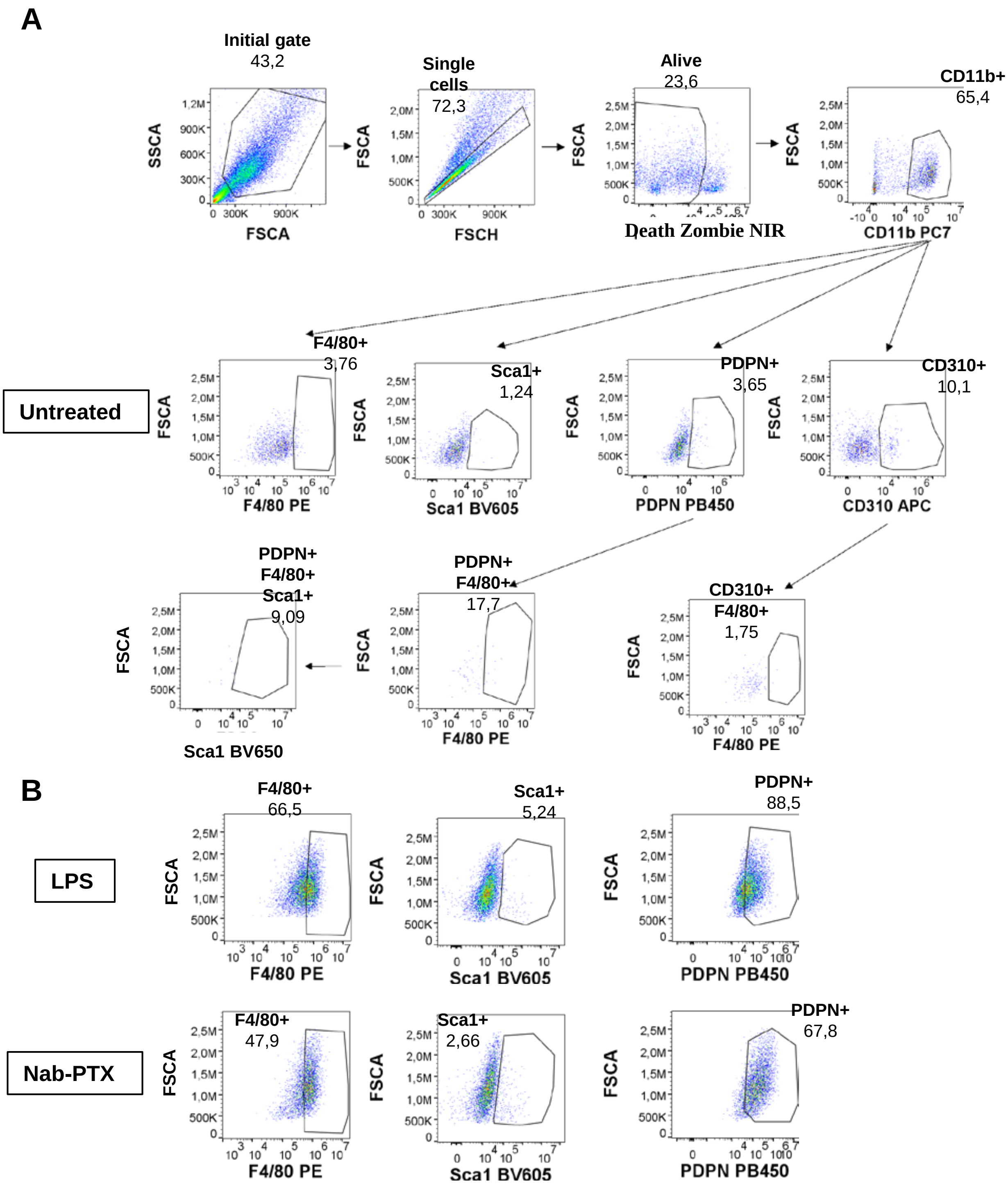

Supplementary Figure 4 . Flow cytometry gating strategy for ex vivo MLECP differentiation corresponding to Figure 5C and Figure 6C.

(A) Representative gating strategy for untreated samples starting from total events using FSC-A and SSC-A to define cell populations. Singlets were selected (FSCH vs FSCA), then live cells gated by excluding dead cells (Zombie NIR^{-}). Leukocytes were separated into CD11b+ subsets, including macrophages (F4/80+), progenitors (Sca1+), stromal-like cells (PDPN+), CD310+ populations, and their combinations (e.g., PDPN+F4/80+Sca1+, CD310+F4/80+). **(B)** Induction of M-LECP markers (CD11b+ F4/80+, CD11b+ Sca1+, CD11b+ PDPN+) after LPS or nab-PTX treatment. Percentages represent frequencies within the parental population.

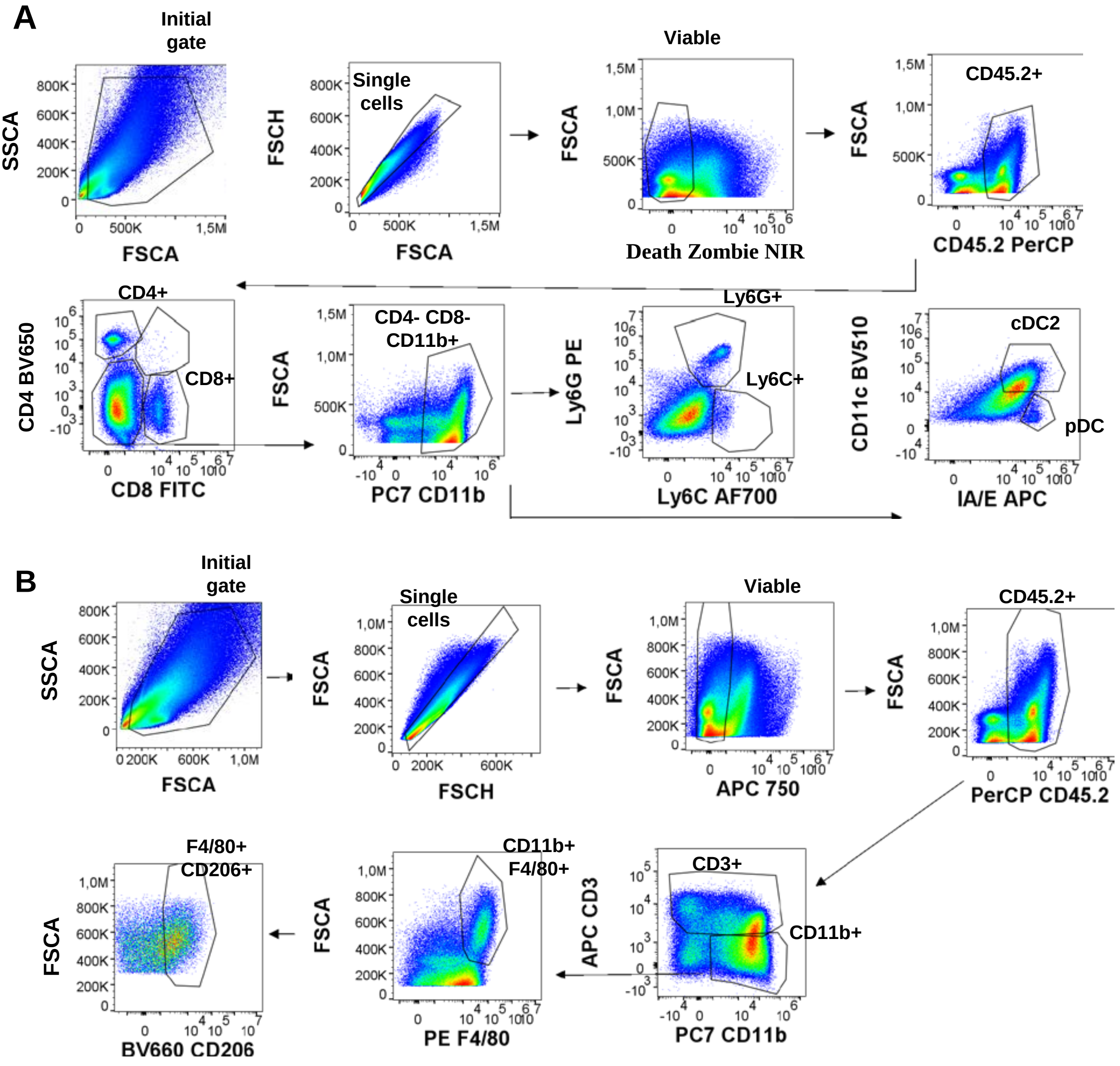

Supplementary Figure 5. Representative flow cytometry gating strategy for Figure 6. **(A)** Gating strategy for identification of lymphoid and myeloid populations using antibody panel 1. Sequential gates select total cells, singlets, live cells (Zombie NIR$^-$), and CD45.2$^+$ leukocytes. Subsequent gating identifies CD4$^+$ and CD8$^+$ T cells, type 2 dendritic cells (cDC2: CD4$^-$ CD8$^-$ CD11b$^+$ CD11c$^+$) , plasmacytoid dendritic cells (pDCs: CD4$^-$ CD8$^-$ CD11b$^-$ CD11c$^+$), CD4$^-$ CD8$^-$ CD11b$^+$ myeloid cells, CD11b$^+$ Ly6G$^+$ granulocytes (CD4- CD8- CD11b+ Ly6G+), and CD11b$^+$ Ly6C$^+$ monocytes (CD4- CD8- CD11b$^+$ Ly6C$^+$). **(B)** Gating strategy for macrophage and myeloid cell phenotyping with antibody panel 2. After selecting live singlet CD45.2$^+$ cells, gates identify CD3- CD11b$^+$ F4/80$^+$ macrophages positive for CD206, as well as CD3$^+$ T cells. Data shown correspond to a representative control tumor sample.

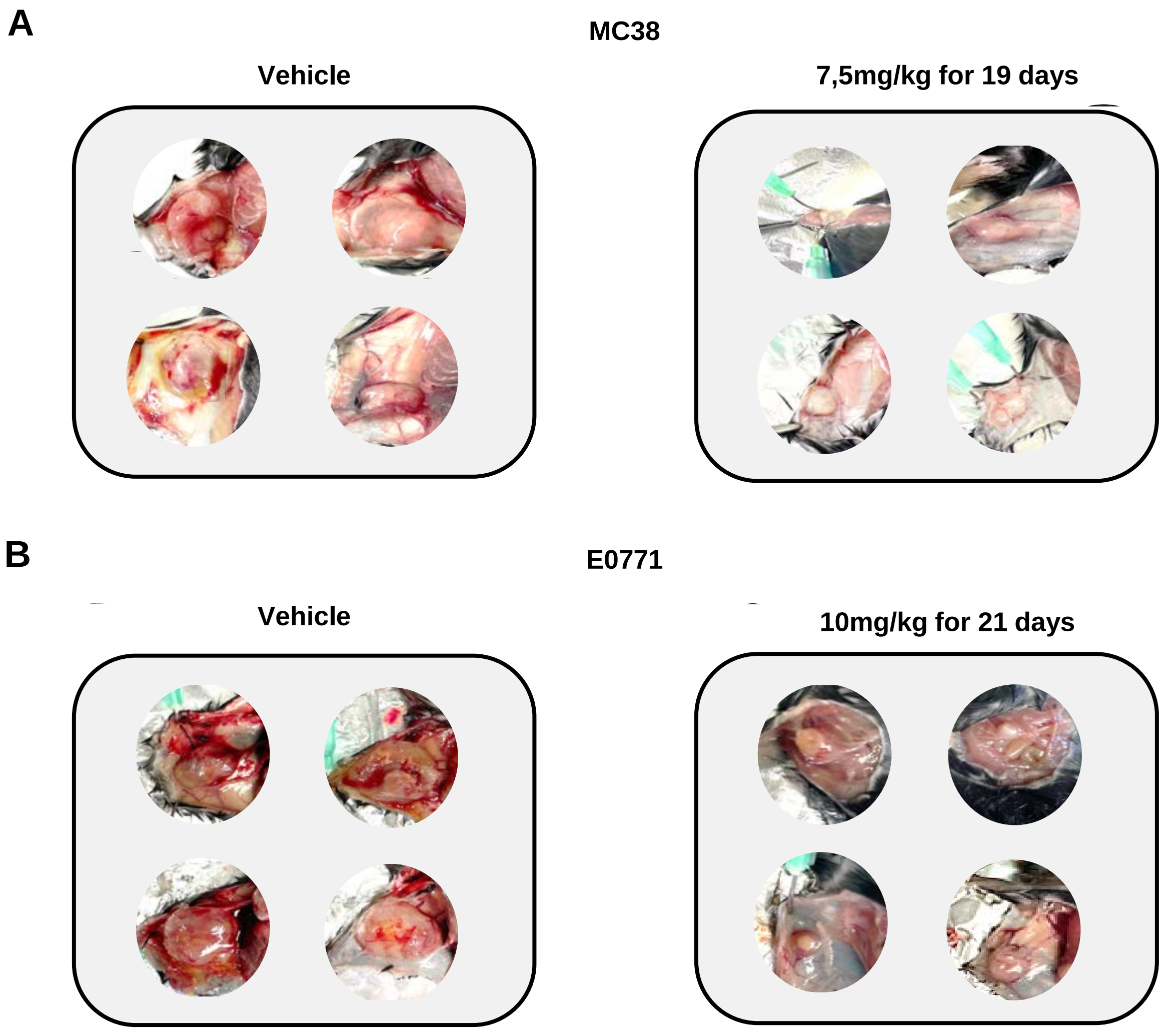

Supplementary Figure 6. Fruquintinib reduces peritumoral vascularization in MC38 and E0771 tumor models.

Representative photographs of MC38 **(A)** and E0771 **(B)** tumor-bearing mice on the day of sacrifice, after daily treatment with vehicle or fruquintinib for 19 days at 7.5 mg/kg (MC38) or 21 days at 10 mg/kg (E0771).

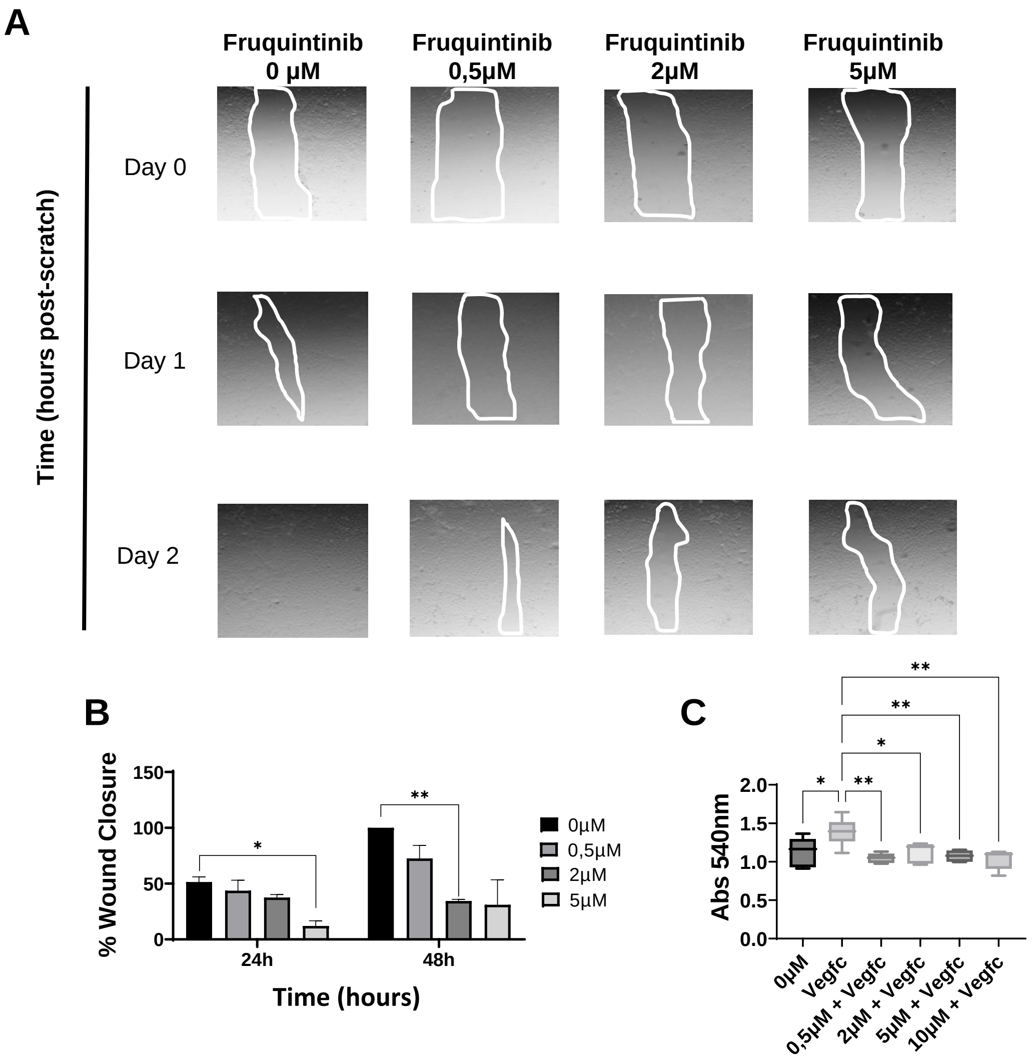

Supplementary Figure 7. Fruquintinib anti-migratory and anti-proliferative effect on lymphatic endothelial SVEC 4-10 cells.

(A-B) Scratch assay on SVEC4-10 cell line under fruquintinib influence. SVEC4-10 cells were serum starved overnight and seeded on next day at a density of 5.10^4 cells per 24 well on DMEM medium with 0,1% FBS in the presence or absence o fruquintinib (0.5µM, 2µM and 5µM) (*n*=3 independent experiments on each group). **(A)** Representative light microscope images at each time point and treatment condition. Black lines indicate the edges of the scratch wound. **(B)** The graph represents the percentage of wound closure 24 and 48 hours after fruquintinib treatment addition. **(C)** Neutral Red uptake assay to evaluate the anti-proliferative effect of fruquintinib. Cells were seeded at a density of 2.10^4 cells per 96 well and serum starved overnight the next day. The following day, cells were treated with VEGFC (500 ng/ml) in the presence or absence of fruquintinib for 24 hours. Neutral Red uptake was quantified spectrophotometrically at 540 nm. Data are presented as min to max ± mean and SEM of 6 independent experiments as determined by one-way ANOVA versus Vegfc alone. *$p < 0.05$, **$p < 0.01$.

A

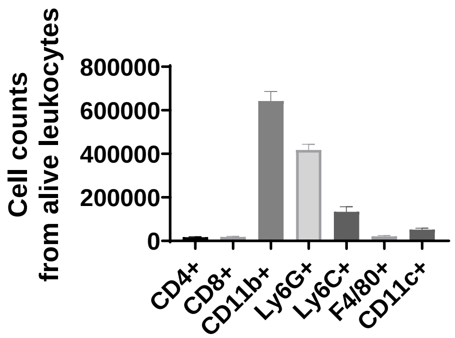

B

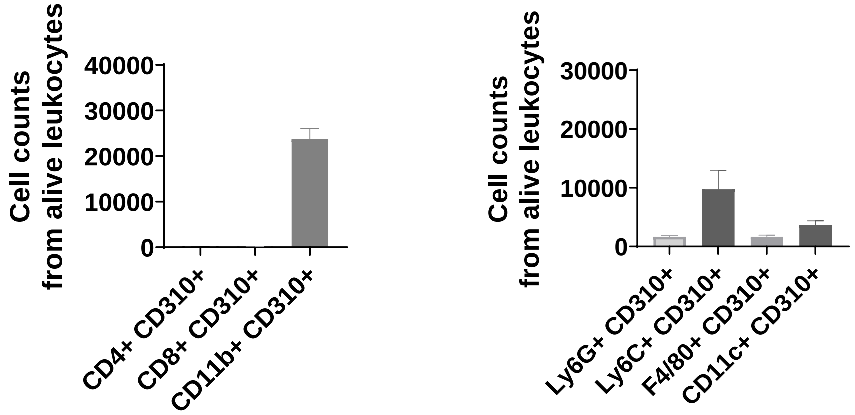

Supplementary figure 8. VEGFR3/FLT4 is expressed in myeloid cells of bone marrow from non-bearing tumor mice.

Cell counts relative to viable leukocytes ($CD45^+$) of $CD4^+$, $CD8^+$ T lymphocytes, $CD11b^+$ myeloid cells, $CD11b^+$ $Ly6C^+$ monocytes, $CD11b^+$ $F4/80^+$ macrophages and $CD11b^+$ $CD11c^+$dendritic cells in bone marrow of non-bearing tumor mice C57BL/6 (A). Cell counts of the same immune cell subsets expressing CD310 (VEGFR3/FLT4) in non-bearing tumor mice (B)

Bone marrow data were obtained from one-tenth of the total marrow cell suspension. Data represent mean ± SEM of two independent experiments with n = 8 mice per group

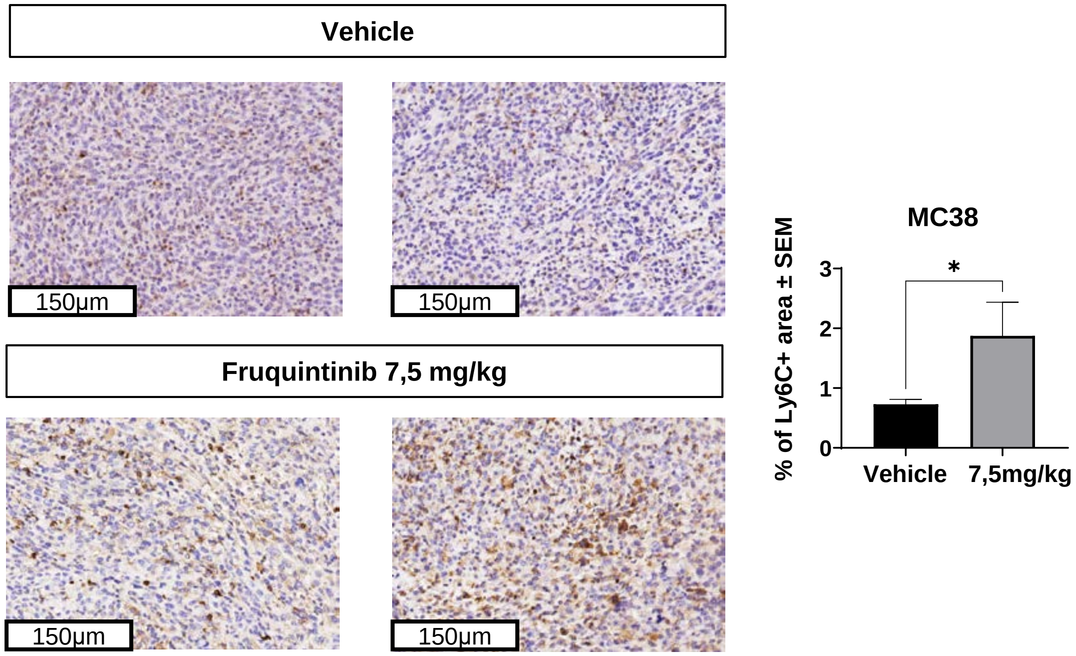

Supplementary Figure 9. Fruquintinib treatment increases Ly6C^{+} monocytes in MC38 tumor-bearing mice.

Representative immunohistochemical staining for Ly6C^{+} monocytes in tumor sections from MC38 tumors, with or without fruquintinib treatment (7.5 mg/kg, 19 days). The right graph shows the percentage of total Ly6C^{+} covered area ($n = 4$ control, $n = 5$ fruquintinib of one independent experiment). Analysis was performed using QuPath version 0.5.1. Data are presented as mean ± SEM. Statistical significance was determined using an unpaired t-test. *$p < 0.05$.

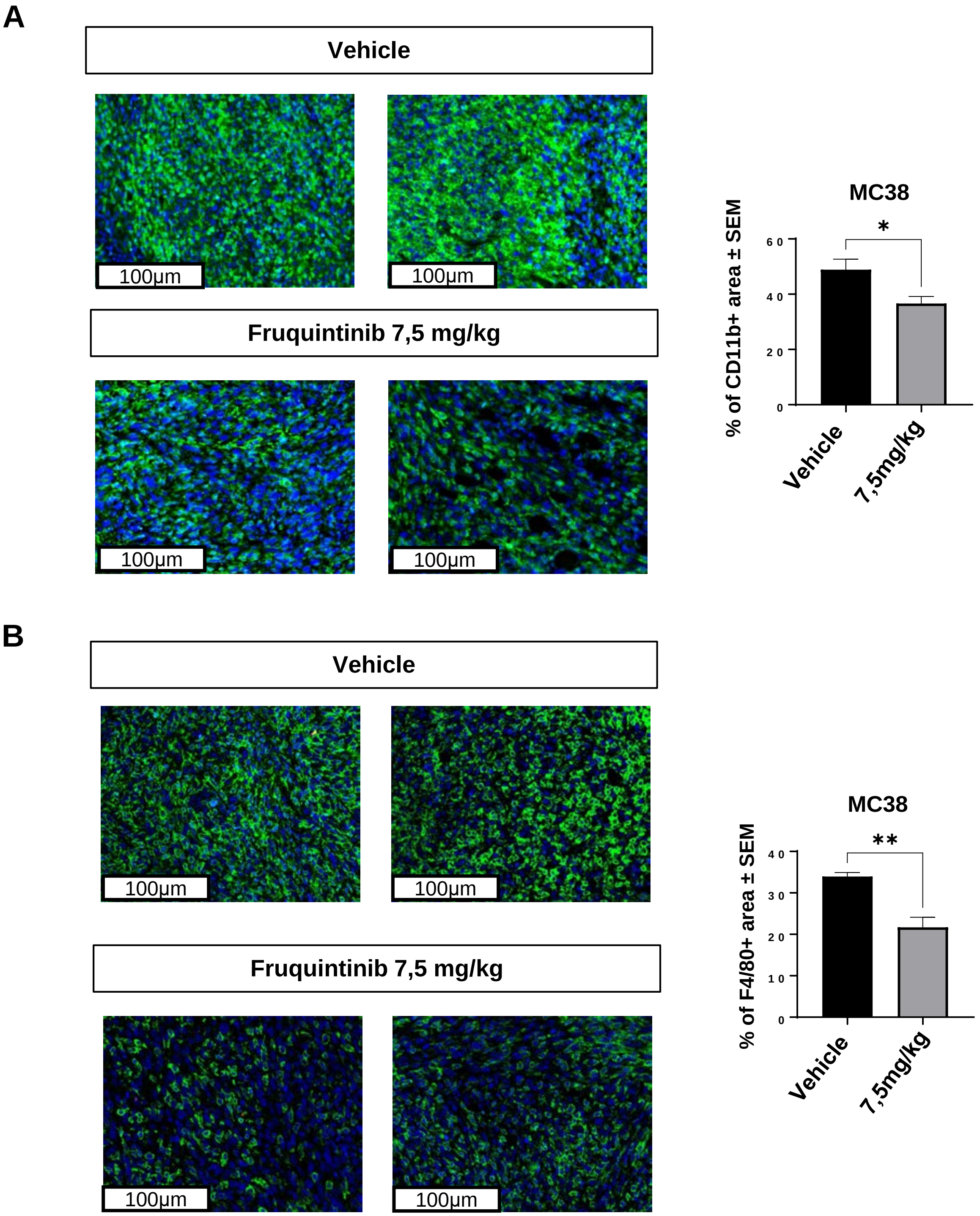

Supplementary Figure 10. Fruquintinib treatment reduces CD11b^{+} myeloid population and F4/80^{+} macrophages in MC38 tumor-bearing mice.

Representative immunofluorescence staining for CD11b^{+} myeloid cells (A) and F4/80^{+} macrophages (B) in tumor sections from MC38 tumors, with or without fruquintinib treatment (7.5 mg/kg, 19 days). Right graphs show the percentages of total CD11b^{+} or F4/80^{+} covered area (n = 5 mice per group of one independent experiment). Analysis was performed using QuPath version 0.5.1. Data are presented as mean ± SEM. Statistical significance was determined using an unpaired t-test. .*$p < 0.05$; . **$p < 0.01$.

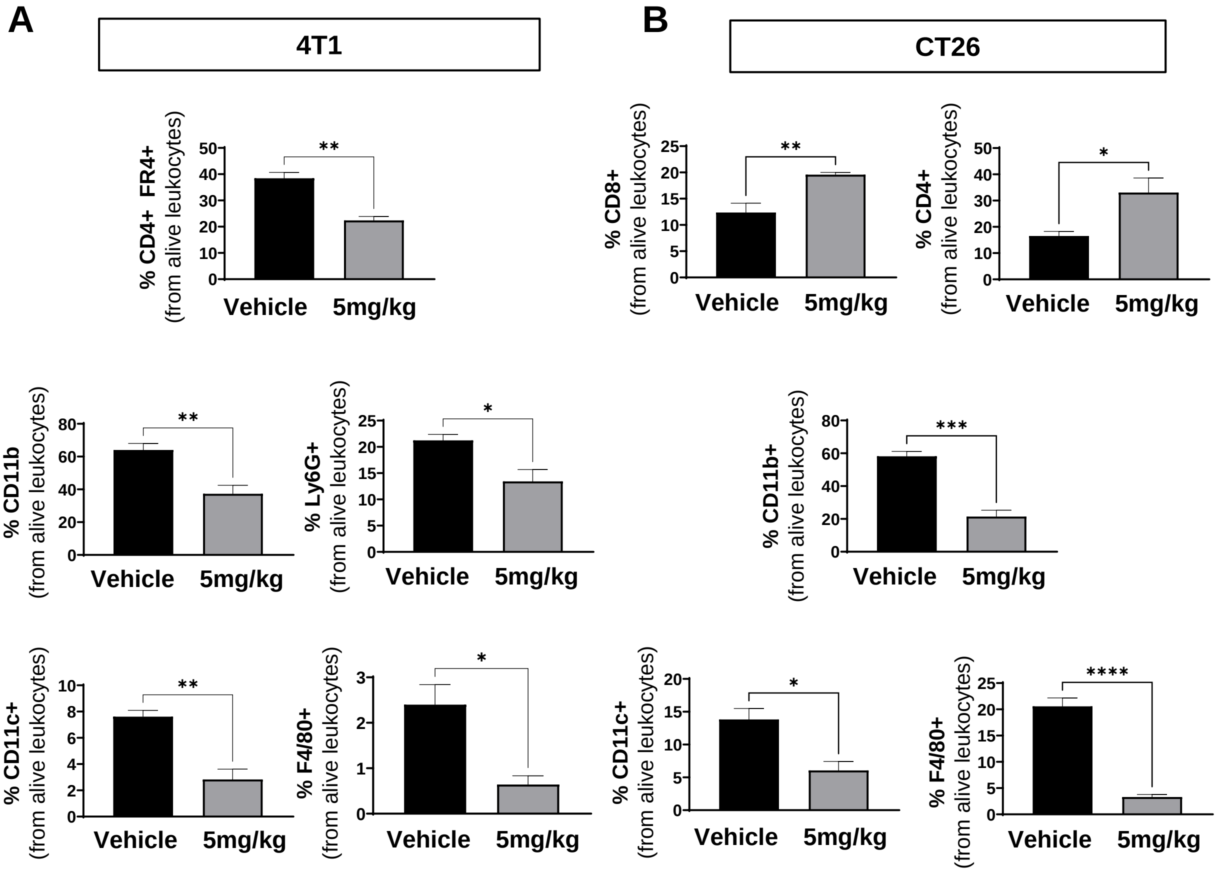

Supplementary figure 11. Fruquintinib treatment modulates immune cell populations in 4T1 and CT26 tumor models.

(A) 4T1 and (B) CT26 tumor-bearing mice were treated orally with vehicle or fruquintinib (5 mg/kg) daily for 15 days. Immune populations were analyzed by flow cytometry from tumor single-cell suspensions. Graphs show frequencies (%) of indicated immune subsets gated on live (Zombie NIR$^-$) CD45.2$^+$ cells: CD4$^+$ regulatory T cells (CD4$^+$FR4$^+$), CD8$^+$ and CD4$^+$ T cells, CD11b$^+$ myeloid cells, Ly6G$^+$ neutrophils, CD11c$^+$ dendritic cells, and F4/80$^+$ macrophages. Gating definitions: CD4$^+$ T cells were identified as CD4$^+$ CD8$^-$; CD8 T cells as CD8$^+$ CD4$^-$; CD11b$^+$ myeloid cells were identified as CD4$^-$ CD8$^-$ CD11b$^+$; Ly6G$^+$ neutrophils as CD4$^-$ CD8$^-$ CD11b$^+$ Ly6G$^+$ Ly6C$^-$; CD11c$^+$ cells as CD45.2$^+$ CD4$^-$ CD8$^-$ CD11b$^+$ Ly6G$^-$ Ly6C$^-$ CD11c$^+$; F4/80$^+$ cells as CD45.2$^+$ CD4$^-$ CD8$^-$ CD11b$^+$ Ly6G$^-$ Ly6C$^-$ F4/80$^+$. Bars represent mean ± SEM. Statistical analysis was performed using an unpaired two-tailed Student's t-test; *$p < 0.05$, **$p < 0.01$, ***$p < 0.001$, ****$p < 0.0001$. n = 4 mice per group for both 4T1 and CT26 models.

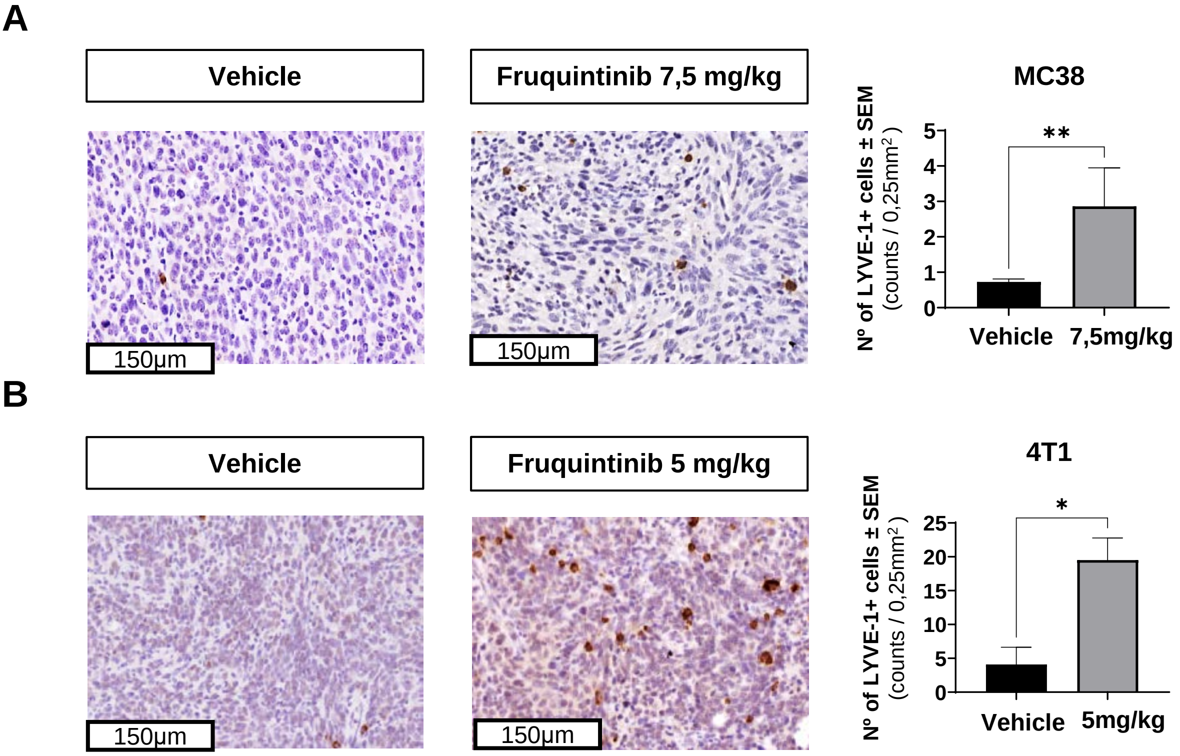

Supplementary Figure 12. Fruquintinib treatment enhances LYVE-1+ precursors in MC38- and 4T1-tumor-bearing mice.

Representative immunohistochemical staining for LYVE-1^+ cells in tumor sections from MC38 (A) and 4T1 (B) tumors, with or without daily fruquintinib treatment (A: 7.5 mg/kg, 19 days; B: 5 mg/kg, 15 days). The right graph shows the number of LYVE-1^+ cells on each region of $0.25mm^2$ analyzed (MC38: $n = 4$ control, $n = 5$ fruquintinib; 4T1: $n = 4$ control, $n = 5$ fruquintinib). Analysis was performed using QuPath version 0.5.1. Data are presented as mean ± SEM. Statistical significance was determined using an unpaired t-test. $*p < 0.05$
