# Supplementary material for: Fruquintinib saddles tumor immune tolerance by curbing pro-tumoral immature myeloid cell populations
Source: Front Immunol. 2025 Dec 11;16:1699980. doi: 10.3389/fimmu.2025.1699980 (PMC12738944; doi:10.3389/fimmu.2025.1699980)
Supplement: Supplementary file 2 [file DataSheet2.docx]

**Immunofluorescence**

Paraffin-embedded tissue sections (3 µm) were deparaffinized through consecutive alcohol washes. Antigen retrieval was performed using citrate buffer (pH 6.0; C9999, Sigma-Aldrich) in a pressure cooker for 40 minutes.

Sections were blocked with 10% BSA in PBS and incubated overnight at 4 °C with primary antibodies: anti-F4/80 (70076, Cell Signaling; 1:200) and anti-CD11b (HS384103, HistoSure; 1:200), diluted in 1% BSA/PBS. After washing, sections were incubated with donkey anti-rabbit Alexa Fluor 594 secondary antibody (A31573, Molecular Probes; 1:200) for 45 minutes at room temperature in the dark. Nuclei were counterstained with Hoechst, and slides were mounted in PBS/glycerol (1:1).

Imaging was performed using a confocal laser scanning microscope LSM 800 (Carl Zeiss Microscopy GmbH, Jena, Germany), and image analysis was conducted with QuPath software (version 0.5.1).
